# A facile synthetic route to benzimidazolium salts bearing bulky aromatic N-substituents

**DOI:** 10.3762/bjoc.11.182

**Published:** 2015-09-17

**Authors:** Gabriele Grieco, Olivier Blacque, Heinz Berke

**Affiliations:** 1 Department of Chemistry, University of Zurich, Winterthurerstrasse 190, CH-8057 Zurich, Switzerland, Fax: (+41)-1-635-6802

**Keywords:** benzimidazolium salts, bulky ligands, cyclization, ligand design, N-heterocyclic carbenes (NHC), ring formation

## Abstract

An atom-economic synthetic route to benzimidazolium salts is presented. The annulated polycyclic systems: 1,3-bis(2,4,6-trimethylphenyl)-1*H*-benzo[*d*]imidazol-3-ium chloride (**1**-Cl), 1,3-bis(2,6-diisopropylphenyl)-1*H*-benzo[*d*]imidazol-3-ium chloride (**2**-Cl), 1,3-diphenyl-1*H*-benzo[*d*]imidazol-3-ium chloride (**3**-Cl), and 1,3-di(pyridin-2-yl)-1*H*-benzo[*d*]imidazol-3-ium chloride (**4**-Cl) were prepared in a two-step synthesis avoiding chromatographic work-up. In the key step triethyl orthoformate is reacted with the corresponding *N*^1^,*N*^2^-diarylbenzene-1,2-diamines and then further transformed in situ, by alkoxy abstraction using trimethylsilyl chloride (TMSCl), and concomitant imidazole ring closure.

## Introduction

Imidazole-based N-heterocyclic carbenes (NHCs) are stable systems serving as ancillary ligands mainly to construct organometallic complexes. These NHCs are sterically and electronically tunable, strongly binding ligand units in complexes in the search for new materials [1–5] and for catalysts of homogeneous catalysis [6–10]. Besides that NHCs have great potential in organocatalysis [11–15], their electronic properties, σ-donor ability and π-back donation can be tuned and, based on these properties, they possess great electronic flexibility. These ligands or catalysts reached a prominent position in the mentioned fields of chemistry. It is noteworthy that on the material’s side NHC-type carbene–borane adducts and metal complexes of them can also be used as electroluminescent materials (OLEDs) [16–19].

After the discovery of NHCs in 1968 by Wanzlick and Öfele, who isolated stable diamino-substituted carbenes, around 20 years later Arduengo further stabilized these potential ligand groups by embedding them into imidazole rings and increased in addition the steric congestion [20]. It is important to recognize that synthetically the stable forms for handling of imidazole carbenes are imidazolium salts. In imidazolium-based NHC chemistry two synthetic aspects are important: 1) there are several fundamental synthetic routes available that allowed substitution in the 4 and 5 positions of the imidazole ring; and 2) the routes available can be carried out as one-pot reactions [21–22] or two-step preparations [23–24].

Annulated polycyclic NHCs can, however, not always be prepared in straightforward ways, especially when the compounds are designed to bear bulky aromatic moieties as N^1^,N^2^-substituents. As mentioned before the synthetic pre-stages of NHCs are always imidazolium salts, but benzimidazolium salts, which possess two aromatic N^1^,N^2^-substituents are rare. More common are benzimidazolium salts having different N^1^,N^2^-substituents, where one of the N-substituent is an aromatic or a benzylic group and the other substituent an alkyl [25–30] or benzyl [31–32] group. A synthetic strategy for the preparation of sterically demanding monoaryl benzimidazolium salts starts from the corresponding benzimidazoles possessing a bulky aryl group introducing the other bulky N-substituent by means of an N-alkylation [33–37]. Indeed benzimidazolium salts that bear N-alkyl or N-alkenyl substituents can be accessed synthetically by simple routes in comparison with those possessing two N-aromatic substituents.

We therefore sought the preparation of sterically demanding *N*^1^,*N*^2^-benzimidazolium systems as, for instance, those accessed by Chianese and co-workers [38]. But for the access to other aryl-substituted benzimidazolium species we had to face complex synthetic pathways, for which we intended to simplify these as much as possible.

**Figure 1:**
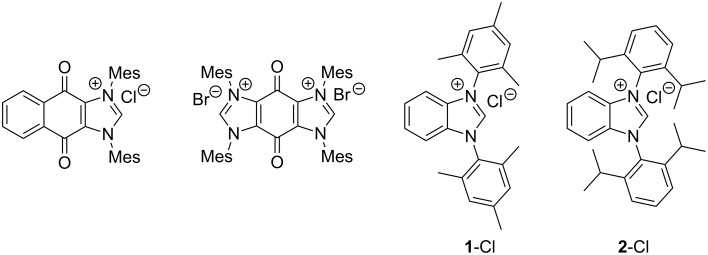
Sterically demanding benzannulated NHCs bearing mesityl rings. From the left side:, 4,9-dihydro-4,9-dioxo-1,3-bis(2,4,6-trimethylphenyl)-1*H*-naphth[2,3-*d*]imidazolium chloride [39], 3,4,5,8-tetrahydro-4,8-dioxo-1,3,5,7-tetrakis(2,4,6-trimethylphenyl)-benzo[1,2-*d*:4,5-*d'*]diimidazolium bromide [41], 1,3-bis(2,4,6-trimethylphenyl)-1*H*-benzo[*d*]imidazol-3-ium chloride (**1**-Cl) [42–43] and 1,3-bis(2,6-diisopropylphenyl)-1*H*-benzo[*d*]imidazol-3-ium chloride (**2**-Cl).

In addition to the 1,3-benzoimidazolium salts prepared by Chianese and co-workers, polycyclic diaminocarbenes were reported possessing mesityl substituents in the N^1^,N^2^-positions and in addition benzoquinone [39–40] or quinone [41] imidazole annulated systems or those with an annulated 1,3-dimesitylbenzene ring (**1**-Cl) [42–43] (Figure 1). The synthetic routes are very sensitive to the respective substitution patterns, for instance, the procedure for **1**-Cl failed to access the analogous 1,3-(2,6-diisopropyl)benzimidazolium salt **2**-Cl.

**Figure 2:**
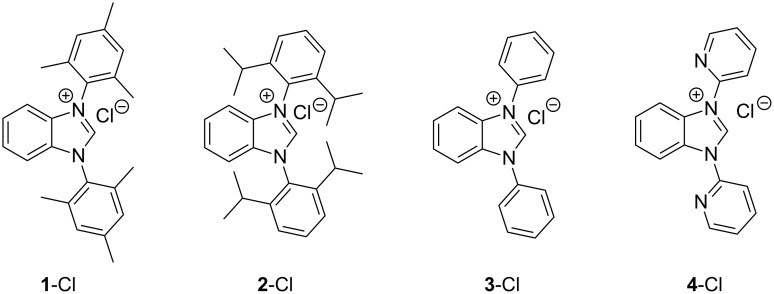
Benzannulated NHCs of this work. Benzannulated imidazolium chloride salts **1**-Cl, **2**-Cl, **3**-Cl and **4**-Cl of various steric demands and ligating properties in complexes.

In this article we describe a two-step synthesis for the sterically hindered benzimidazolium salts: 1,3-bis(2,4,6-trimethylphenyl)-1*H*-benzo[*d*]imidazol-3-ium chloride (**1**-Cl) [42–43], 1,3-bis(2,6-diisopropylphenyl)-1*H*-benzo[*d*]imidazol-3-ium chloride (**2**-Cl), 1,3-diphenyl-1*H*-benzo[*d*]imidazol-3-ium chloride (**3**-Cl) [44–45] and 1,3-di(pyridin-2-yl)-1*H*-benzo[*d*]imidazol-3-ium chloride (**4**-Cl) [45] (Figure 2). The synthesis starts from suitable aryl diamines applying the combination of the reagents trimethylsilyl chloride and triethyl orthoformate as C_1_ component to accomplish concomitantly cyclization to the imidazole rings. We expected high yields and reduced overall reaction times when compared with the previously reported synthetic pathways.

Excluding the strongly sterically encumbered benzimidazolium salt **2**-Cl, the benzannulated NHCs **1**-Cl, **3**-Cl and **4**-Cl were prepared earlier, however, **1**-Cl was prepared by a relatively complex reaction scheme. Moreover, the benzimidazolium salts **1**-Cl to **4**-Cl possessing a variety of N^1^,N^2^-substituents were designed to eventually allow facile release of the benzoimidazole carbenes acting eventually as ligands in complexes by deprotonation avoiding additional complications that could arise from the anions of the benzimidazolium salts.

## Results and Discussion

**Scheme 1:**
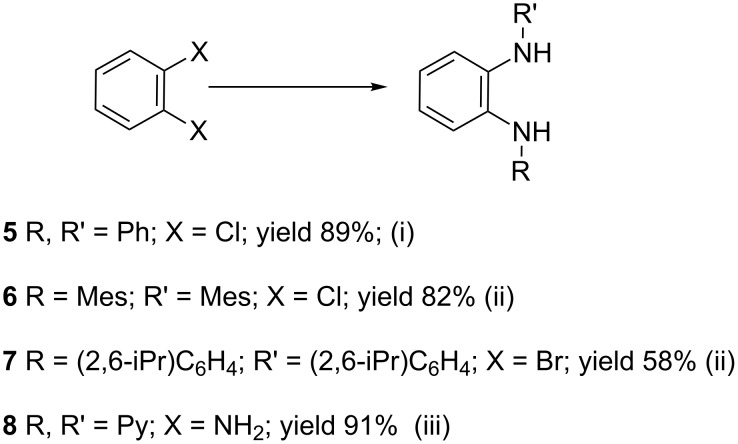
Synthesis of the *N*^1^,*N*^2^-diaryl-1,2-benzenediamines **5**, **6**, **7** and **8**. i) Pd(dba)_2_, P(*t*-Bu)_3_, *t*-BuONa, toluene, 92 °C. ii) Pd(dba)_2_, P(*t*-Bu)_3_, *t*-BuONa, toluene, 115 °C. iii) S_N_Ar reaction: 2-chloropyridine, neat, 185 °C, microwave.

The starting point for the syntheses of the benzannulated NHCs **1**-Cl, **2**-Cl and **3**-Cl was the development of a straightforward palladium-catalyzed preparation of the known *N*^1^,*N*^2^-diphenylbenzene-1,2-diamine compounds **5**, **6** and **7** in dependence on an earlier synthetic route [46] (Scheme 1). In the case of compound **5** a better yield (89%) could be accomplished when the reaction was carried out at 92 °C instead of 120 °C as reported [47].

**Scheme 2:**
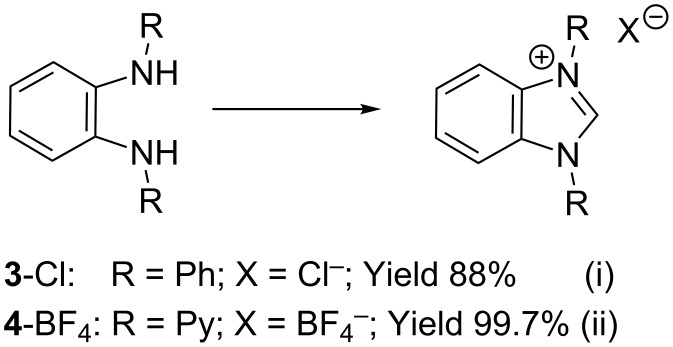
Previous synthesis of the benzannulated NHCs **3**-Cl and **4**-BF_4_. Ring closure. i) (EtO)_3_CH, HCl (conc.), HCOOH, 80 °C [44]. ii) Microwave assisted synthesis: NH_4_BF_4_, (EtO)_3_CH, 160 °C [47].

Somewhat modified approaches were used for the syntheses of the *N*^1^,*N*^2^-diarylbenzene-1,2-diamines **6** and **7** compared with the preparation of the *N*^1^,*N*^2^-di(pyridine-2-yl)benzen-1,2-diamine (**8**). The Buchwald–Hartwig amination was applied in the syntheses of **5** and **6** where 1,2-dichlorobenzene was coupled with aniline and 2,4,6-trimethylaniline, respectively (Scheme 1). Attempting the synthesis of the *N*^1^,*N*^2^-bis(2,6-diisopropylphenyl)benzene-1,2-diamine (**7**) and using 1,2-dichlorobenzene as the starting compound, the mono-substituted product was obtained. To avoid this complication 1,2-dibromobenzene had to be applied to eventually approach the preparation of **7**. For **8** a reported synthetic procedure was used [48–49] consisting of nucleophilic aromatic substitutions (S_N_Ar) of benzene-1,2-diamine at 2-chloropyridine (Scheme 1). Once all the different *N*^1^,*N*^2^-diarylbenzene-1,2-diamines were prepared a method had to be developed to build up the imidazolium salts by ring closure. **3**-Cl and **4**-Cl could principally be obtained from the corresponding *N*^1^,*N*^2^-diarylbenzene-1,2-diamines via two different cyclization pathways leading to the benzimidazolium salts **3**-Cl [44] and **4**-BF_4_ [48] (Scheme 2). But we also planned to access the chloride compounds **1**-Cl, **3**-Cl and **4**-Cl reported previously as tetrafluoroborate [48] and chloride [45] salts for two reasons: we wished to find a faster way to access them and it seemed advantageous to aim preferentially at the synthesis of benzimidazolium chlorides than at [BF_4_]^−^ salts, since in case the carbene will be generated in “in situ” reactions in the presence of metal complexes, the lower stability of the [BF_4_]^−^ anion could lead to complications generating side-products [50–52]. The preparations of both **3**-Cl and **4**-Cl started along the given lines with the syntheses of the respective aryl-substituted diamines, which had to undergo ring closure in the presence of a C_1_ component. For **3**-Cl and **4**-Cl the synthetic procedures proceed without noticeable problems, while complications were faced in the synthesis for **1**-Cl and **2**-Cl, particularly during the benzimidazole ring closures. Excluding the procedure of Borguet [42–43] all the known procedures to accomplish imidazole ring formation [44–45,48,53–54] of either **6** and **7** failed.

For instance, the method of Hintermann [23] that uses the combination of paraformaldehyde and trimethylsilyl chloride (TMSCl) gave satisfying results in the preparation of the known IMes and IPr derivatives, but failed for the synthesis of both **1**-Cl and **2**-Cl. In fact there are examples of *N*^1^,*N*^2^-bisarylethandiamines, which could be cyclisized to benzimidazolium salts in the presence of air, paraformaldehyde and hydrochloric acid [55], and the success of this synthetic approach was presumably crucially depending on the mode of action of O_2_ oxidizing the aminal intermediate. The failures to access **1**-Cl and **2**-Cl may originate from the high steric hindrance of the 2,6-substituents of the *N*^1^,*N*^2^-diarylbenzene-1,2-diamines preventing initial aminal formation. Involving instead triethyl orthoformate has the advantage that this C_1_ building block provides the right oxidation state for the cyclization process making the involvement of an oxidizing agent unnecessary [23] and, moreover, it possesses high electrophilicity required for this reaction. The method of Chianese et al. [38] demonstrated that the cyclization of *N*^1^,*N*^2^-diarylbenzene-1,2-diamine can be achieved with bulky substituents in the 3-, 4- and 5-positions of the *N*^1^,*N*^2^-phenyl rings, but this study clearly showed also that cyclization of the diarylbenzene diamines get difficult when bulkier groups are in 2- and 6-position of the *N*^1^,*N*^2^-substituents.

**Scheme 3:**
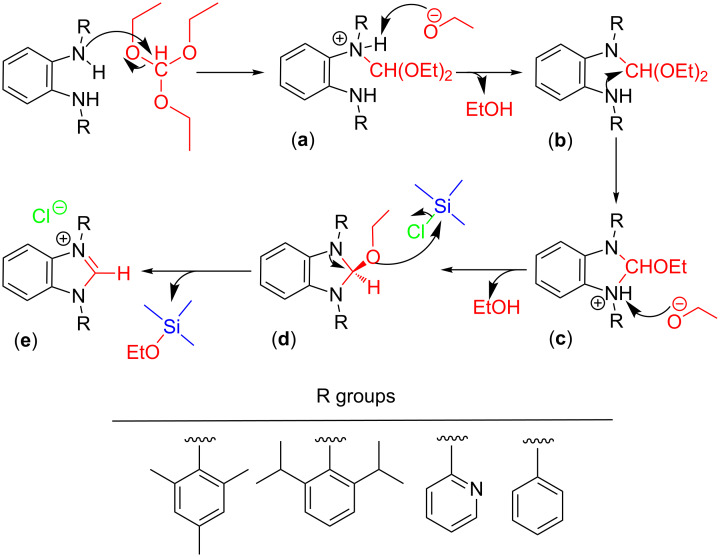
Proposed ring-closure mechanism for **1**-Cl, **2**-Cl, **3**-Cl and **4**-Cl.

A reaction course is proposed for the ring closure of **5**, **6**, **7** and **8** forming the benzimidazolium salts **1**-Cl, **2**-Cl, **3**-Cl and **4**-Cl, which is suggested to pass through stages **a** and **b** with alcohol elimination (Scheme 3) and eventually then cyclization is initiated via the 2-ethoxy-1,3-diaryldihydrobenzimidazole species **c** and **d** enforced by the applied higher temperatures. TMSCl is assumed to abstract an alkoxide group and to deliver at the same time the chloride as the preferred counterion for the imidazolium salts.

**Figure 3:**
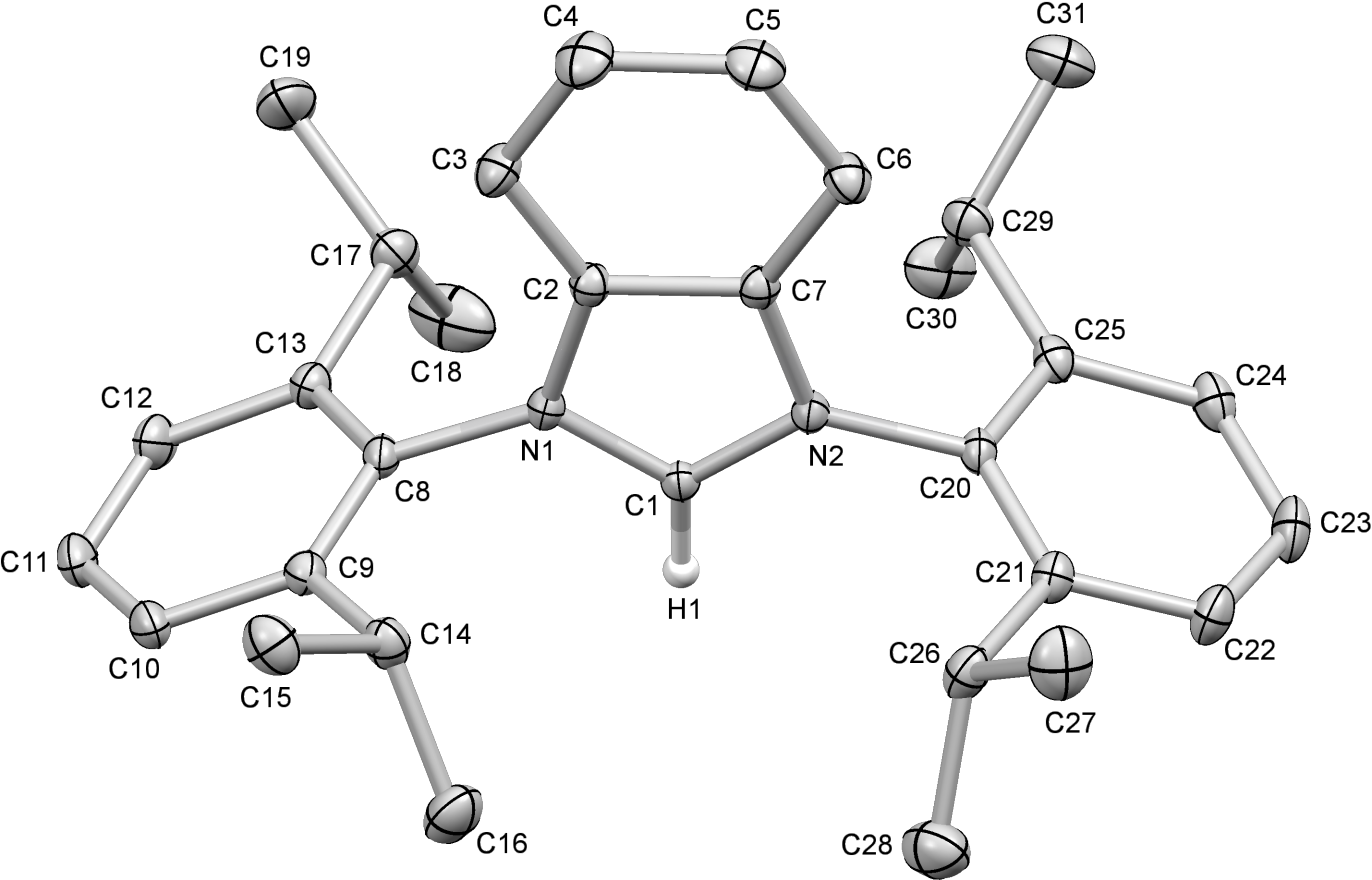
Molecular structure of **2**-Cl. The solvate molecule, the counterion and the hydrogen atoms are omitted for clarity. The displacement ellipsoids are drawn at the 30% probability. Selected bond lengths (Å) and angles (°): N2–C1–N1 109.3(2), N2–C1–H1 126.1(17), N1–C1–H1 124.6(17), N1–C1 1.334(3), N2–C1 1.330(3), N1–C2 1.392(3), N2–C7 1.397(3), N1–C8 1.448(3), N2–C20 1.447(3), C1–H1 0.97(3).

As said, temperature plays a decisive role in the formation of stage **d**, requiring the *N*,*N*’-diarylbenzene-1,2-diamines to be heated in triethyl orthoformate at almost reflux temperature (145 °C) for reaction times between 10 and 20 min. Then TMSCl was added in large excess all at once leading to precipitation of greyish products, which indicated the formation of the benzimidazolium chlorides. In this way not only the known compound **1**-Cl could be accessed in a short total reaction sequence (2 steps, one isolated intermediate product) instead of the 4 steps of the synthetic route reported earlier [42–43] (Table 1). The application of new reagents avoided at the same time tedious column chromatography. Even the more sterically encumbered and elusive compound **2**-Cl could obtained in this way (X-ray diffraction structure displayed in Figure 3). The yields of **1**-Cl and **2**-Cl were 68% and 28%, respectively.

**Table 1:** Comparison of various preparations of the benzannulated NHCs: **1**-Cl, **2**-Cl, **3**-Cl and **4**-Cl, and of **1**-BF_4_, **3**-BF_4_ and **4**-BF_4_.

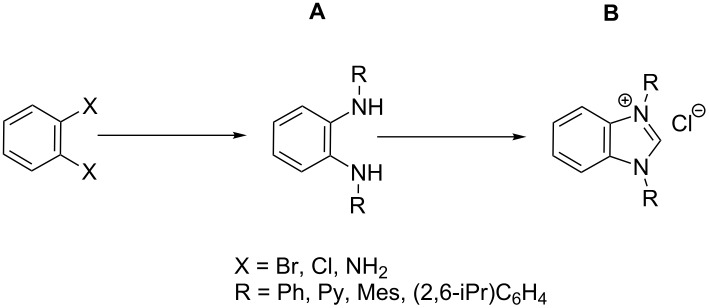								

Starting materials	Reaction conditions	Intermediate product (**A**)	Reaction conditions	Final product (**B**)	Time (h)^a^	Steps^b^	Overall yield (%)	Ref.

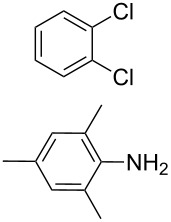	115 °C, 15 h, Pd(dba)_2_, P(*t*-Bu)_3_, *t*-BuONa, toluene yield 82%	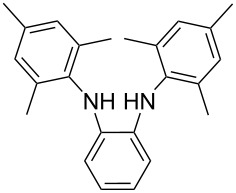 **6**	145 °C, 1,25 h, HC(OEt)_3_, neat, TMSCl yield 67%	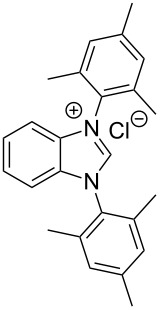 **1-**Cl	16.25	2	55	This work
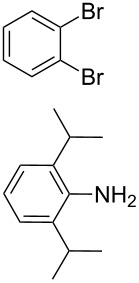	115 °C, 15 h, Pd(dba)_2_, P(*t*-Bu)_3_, *t*-BuONa, toluene yield 58%	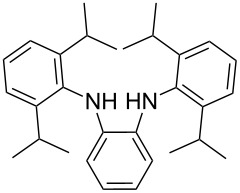 **7**	145 °C, 3.3 h, HC(OEt)_3_, neat, TMSCl yield 28%	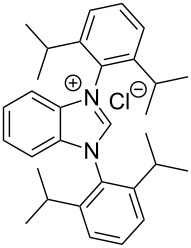 **2-**Cl	18.3	2	16	This work
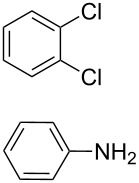	92 °C, 4 h, Pd(dba)_2_, P(*t*-Bu)_3_, toluene yield 89%	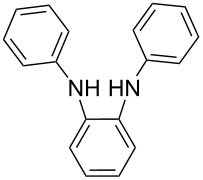 **5**	145 °C, 1 h, HC(OEt)_3_, neat, TMSCl Yield 83%	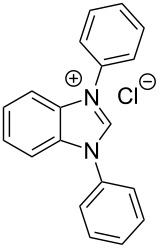 **3-**Cl	5	2	74	This work
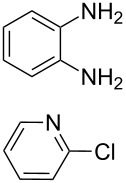	Microwave, 185 °C, 0.58 h, neat, yield 91%	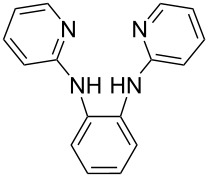 **8**	145 °C, 1 h, HC(OEt)_3_, neat, TMSCl yield 98%	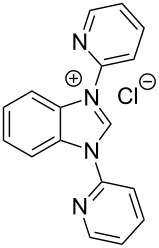 **4-**Cl	1.75	2	89	This work^c^
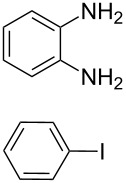	80 °C, 18 h, DMSO, CuI, L-Pro, K_2_CO_3_ yield 30%	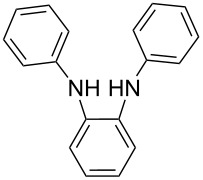 **5**	80 °C, 2 h, HC(OEt)_3_, HCl conc., HCOOH cat. yield 91%	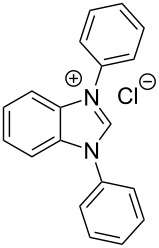 **3-**Cl	20	2	27	[44]
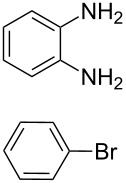	110 °C, 15 h, Pd_2_(dba)_3_, BINAP, NaO*t*-Bu, toluene yield 86%	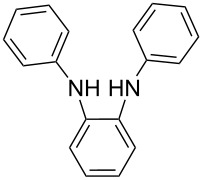 **5**	80 °C, 2 h, HC(OEt)_3_, HCl conc., HCOOH cat. yield 90%	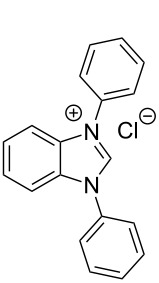 **3-**Cl	17	2	77	[45]
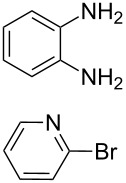	110 °C, 15 h, Pd_2_(dba)_3_, BINAP, NaO*t*-Bu, toluene yield 91%	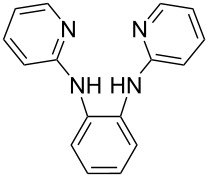 **8**	80 °C, 2 h, HC(OEt)_3_, HCl conc., HCOOH cat. yield 94%	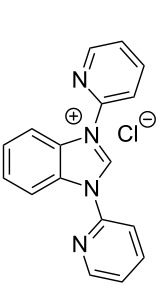 **4-**Cl	17	2	85	[45]
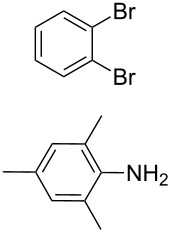	110 °C, 14 h, Pd(OAc)_2_, BINAP, NaO*t*-Bu, toluene yield 86%	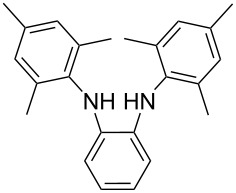 **6**	three steps^d^ total time 97 h overall yield 29%	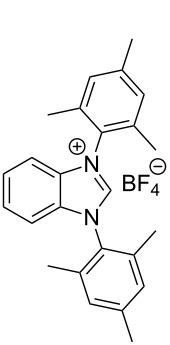 **1-**BF_4_	111	4	25	[42–43]
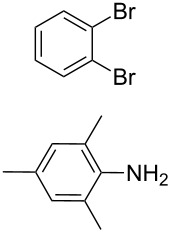	60 °C, 15 h, Pd(OAc)_2_, BINAP, NaO*t*-Bu, toluene yield 86%	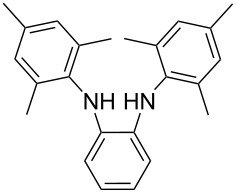 **6**	−78 °C, THF, 12-crown-4, *n*-BuLi, TMSCl, Cr(CO)_6_ yield 13%	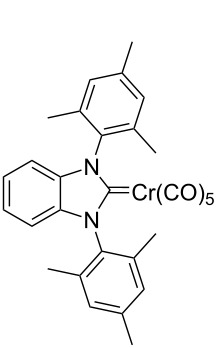	17	2	11	[53]^e^
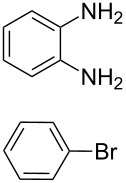	110 °C, 5 h, Pd_2_(dba)_3_, Xantphos, NaO*t*-Bu, toluene yield 91%	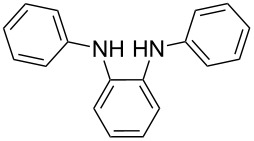 **5**	145 °C, 18 h, neat, NH_4_BF_4_, HC(OEt)_3_ yield 91%	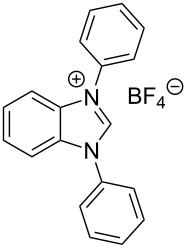 **3-**BF_4_	23	2	82	[54]
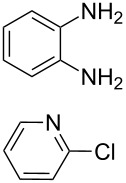	microwave, 185 °C, 0.58 h, neat, yield 91%	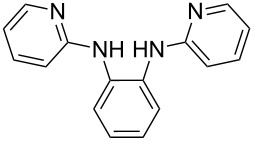 **8**	microwave, 160 °C, 0.75 h, neat, NH_4_BF_4_, HC(OEt)_3_ yield 99%	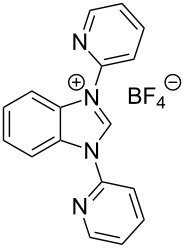 **4-**BF_4_	2	2	90	[48]

^a^Total reaction time: The overall reaction times refer to reaction times and do not take into account the work-up times needed in order to obtain either the intermediate and final products. ^b^Total number of steps: Steps with isolated intermediate or final products. ^c^Compound **8**, precursor to **4**-Cl or **4-**BF_4_, was prepared according to Ref. [47]. ^d^1^st^ step: NaIO_4_, SiO_2_, CH_2_Cl_2_/H_2_O, 24 h, room temperature, Yield 90%; 2^nd^ step: a) PivOCH_2_Cl, AgOTf, KOAc, CH_2_Cl_2_, 48 h, 50 °C. b) PivOCH_2_Cl, AgOTf, 24 h, 50 °C (further 0.5 equiv of the pivalate/silver salt solution); 3^rd^ step: HBF_4_, H_2_O, 1 h, room temperature, yield 32% (over two steps). ^e^The intermediate **6** reacts with a carbamoyl chromate intermediate to give rise directly to the organometallic complex (1,3-bis(2’,4’,6’-trimethylphenyl)benzimidazol-2-ylidene)pentacarbonyl chromium(0).

The previous synthesis of **1**-Cl by Borguet and co-workers [42] required 4 steps. All the other earlier preparations and the preparation of this paper shown in Table 1 were accomplished in two steps. The overall reaction time required for the earlier synthetic access of **1**-Cl was quite high (112 h), as well, in comparison with the use of the TMSCl–triethyl orthoformate reagent presented in this work. By this the total reaction time could be shortened to 17 h and the overall yields raised from 23% to 55%. Despite the fact that 2-step syntheses are available for **3**-Cl and **4**-Cl, Table 1 suggests that the introduction of the TMSCl–triethyl orthoformate reagent has advantages in terms of overall yields and reaction times. The overall achieved yield of 16% for **2**-Cl is indeed low, but the short overall reaction time (19 hours) and the low number of steps (2) together with the fact that this is the only way to access this compound, makes the given route an acceptable synthetic pathway.

## Conclusion

We presented a new synthetic route for the synthesis of benzannulated imidazolium salts bearing bulky aromatic N-substituents. The synthetic approach applied allowed to prepare the as yet inaccessible 1,3-diisopropylphenylbenzimidazolium chloride **2**-Cl in two steps starting from 1,2-halo-substituted benzenes avoiding purification by column chromatography. In addition the known 1,3-dimesitylbenzimidazolium chloride **1**-Cl could be synthesized in better yields and much shorter reaction times when compared with the previously reported method. The known benzannulated NHCs 1,3-diphenylbenzimidazolium chloride **3**-Cl and the 1,3-di(pyridin-2-yl)benzimidazolium chloride **4**-Cl could be prepared also in excellent overall yields and reduced reaction times, if compared with the synthetic access by previous methods. The synthetic access presented in this paper possesses various advantages over the conventional methodologies for the synthesis of benzimidazolium salts with bulky N-substituents. The developed synthetic route may show greater generality and therefore constitutes a valid alternative to the earlier synthetic accesses.

## Experimental

### X-ray diffraction study

Crystallographic data for the structure of **2**-Cl has been deposited with the Cambridge Crystallographic Data Centre as supplementary publication number CCDC 1018301. Copies of the data can be obtained free of charge, on application to CCDC, 12 Union Road, Cambridge CB2 1EZ, UK (e-mail: deposit@ccdc.cam.ac.uk).

**2-Cl:** Crystal data for C_32_H_41_N_2_Cl_3_ (*M* = 560.02 g/mol): monoclinic, space group *P*2_1_/n (no. 14), *a* = 12.6905(3) Å, *b* = 18.5911(3) Å, *c* = 14.3067(4) Å, β = 113.687(3)°, *V* = 3091.02(15) Å^3^, *Z* = 4, *T* = 183(2) K, μ(Cu K_α_) = 2.843 mm^−1^, *D*_calc_ = 1.203 g/cm^3^, 40306 reflections measured (9.5° ≤ 2Θ ≤ 178.9°), 7060 unique (*R*_int_ = 0.0323, *R*_sigma_ = 0.0199) which were used in all calculations. The final *R*_1_ was 0.0777 (I > 2σ(I)) and *wR*_2_ was 0.2142 (all data).

### General procedures

All manipulations were carried out under an atmosphere of dry nitrogen using standard Schlenk techniques or in a glove box (M. Braun 150B-G-II) filled with dry nitrogen. Solvents were freshly distilled under N_2_ by employing standard procedures and were degassed by freeze–thaw cycles prior to use. All chemicals used were purchased from Sigma-Aldrich and used as received. The deuterated solvents were dried with sodium/benzophenone and vacuum transferred for storage in Schlenk flasks fitted with Teflon valves. ^1^H NMR, ^13^C{^1^H} NMR data were recorded on a Varian Gemini-300, a Varian Mercury 200 or Bruker DRX 500 spectrometer using 5 mm diameter NMR tubes. Chemical shifts are expressed in parts per million (ppm). ^1^H and ^13^C{^1^H} NMR spectra were referenced to the residual proton or ^13^C resonances of the deuterated solvent. Signal patterns are reported as follows: s, singlet; d, doublet; dd, doublet of doublets; t, triplet; m, multiplet, q, quartet. ESIMS spectrometric data were obtained from an HCT Esquire Bruker Daltonics instrument, while the EIMS spectrometric data were obtained from a Varian 450GG-Saturn 2000 GC/MS/MS instrument using a Brechbühler column (30 m, ZB-5ms). The reactions that required the use of a microwave reactor were carried out using an Anton Paar Monowave 300. The high resolution ESI were performed by the Mass Service of the University of Zurich using a Bruker maXis apparatus. The compounds **5** [44], **6** [52], **7** [46], **8** [47–48] were prepared according to literature with small modifications.

**Synthesis of 1,3-bis(2,4,6-trimethylphenyl)-1*H*-benzo[*d*]imidazol-3-ium chloride (1-Cl).** In a 125 mL two neck round-bottomed flask was weighed *N*^1^,*N*^2^-bis(2,4,6-trimethylphenyl)-1,2-benzenediamine (**6**, 600 mg, 1.74 mmol) and triethyl orthoformate (60 mL) was added and a fractional distillation apparatus equipped with a Vigreux column and a thermometer on the head of the latter was connected with the central neck of the flask. The green solution was stirred at 145 °C until the color switched to orange (15 min) and then a small flux of nitrogen was passed through the solution until 30 mL of a mixture of EtOH and HC(OEt)_3_ distilled out (1 hour). Then the temperature was lowered to 90 °C and trimethylsilyl chloride (10 mL, 76.43 mmol) was added at once. A white-grey solid started to form after 10 minutes. Then the solution was cooled down to room temperature and the precipitate was filtered off and washed with Et_2_O (3 × 15 mL) then dried to afford in 455 mg of **1-Cl** as an off-white powder (1.16 mmol, 390.95 g/mol). Yield 67%. ^1^H NMR (CD_3_OD, 300 MHz) δ 10.09 (s, 1H, N-CH1-N), 7.84 (dd, *J* = 3.2 Hz, *J* = 6.3 Hz, 2H, H-4, H-5), 7.56 (dd, *J* = 3.2 Hz, *J* = 6.3 Hz, 2H, H-3, H-6), 7.30 (s, 4H, H-10, H-12, H-16, H-18), 2.45 (s, 6H, p-CH_3_, mesityl), 2.11 (s, 12H, o-CH_3_, mesityl); ^13^C{^1^H}(CD_3_OD, 75 MHz) δ 143.55 (N-C1-N), 136.54 (N-C2=C7-N), 132.94 (C4=C5), 131.27 (N-C8, C11), 130.24 (C3, C6), 129.34 (C10, C12), 114.93 (C9, C13), 21.34 (p-CH_3_), 17.45 (o-CH_3_); HRMS–ESI (MeOH): calcd for C_25_H_27_N_2_^+^, 355.21688; found, 355.21644.

**Synthesis of 1,3-bis[2,6-bis(1-methylethyl)phenyl]-1*H*-benzo[*d*]imidazol-3-ium chloride (2-Cl).** In a 50 mL two neck round-bottomed flask was weighed *N*^1^,*N*^2^-bis[2,6-bis(1-methylethyl)phenyl]-1,2-benzenediamine (**7**, 100 mg, 0.23 mmol) and triethyl orthoformate (20 mL) was added and a fractional distillation apparatus equipped with a Vigreux column and a thermometer on the head of the latter was connected with the central neck of the flask. The colourless suspension was stirred at 145 °C then after 20 minutes the colour switched to green. A small flux of nitrogen was passed through the solution until 15 mL of a mixture of EtOH and HC(OEt)_3_ distilled out (40 min). Then fresh triethyl orthoformate (3 mL) was added to the solution, followed by the trimethylsilyl chloride (4 mL, 31.51 mmol) added at once. The solution, that switched from dark green to dark red, was stirred at 50 °C for 3 hours, then the solvent was removed and the red solid was triturated with Et_2_O (20 mL) and the precipitate was filtered off, washed with further Et_2_O (3 × 10 mL), then triturated with acetone (3 mL) and finally dried to afford 31 mg of **2**-Cl as a grey powder (0.065 mmol, 475.11 g/mol). Yield 28%. ^1^H NMR (CD_2_Cl_2_, 300 MHz) δ 12.95 (s, 1H, N-C*H*1-N), 7.74 (m, 6H, H-10, H-11, H12, H14, H-15, H-16), 7.52 (d, *J* = 7.8 Hz, 2H, H-3, H-6), 7.39 (dd, *J* = 3.2 Hz, *J* = 6.3 Hz, 2H, H-4, H-5), 2.29 (sept., *J =* 9Hz, 4H, CH_3_-C*H*-CH_3_ isopropyl), 1.33 (d, *J =* 9 Hz, 12H, C*H_3_*-isopropyl), 1.17 (d, *J =* 9 Hz, 12H, C*H_3_*-isopropyl); ^13^C NMR (CDCl_3_, 75 MHz) δ 146.80 (N-C1-N), 146.02 (N-C8), 132.21 (N-C2=C7-N), 128.5 (C9, C13, C15, C19), 127.45 (C10, C12, C16, C18), 124.97 (C11, C17), 113.42 (C4=C5), 29.42 (CH_3_-*C*H-CH_3_, isopropyl), 24.73 (CH_3_, isopropyl), 23.10 (CH_3_, isopropyl); HRMS–ESI (MeOH): calcd for C_31_H_39_N_2_^+^, 439.31078; found, 439.31042.

**Synthesis of 1,3-diphenylbenzimidazolium chloride (3-Cl).** In a 50 mL two neck round-bottomed flask was weighed *N*,*N*’-diphenylbenzene-1,2-diamine (**5**, 50 mg, 0.19 mmol) and triethyl orthoformate (15 mL) was added and a fractional distillation apparatus equipped with a Vigreux column and a thermometer on the head of it, was connected with the central neck of the flask. The light blue suspension was stirred at 145 °C until the suspension became a light green solution (20 min), then a small flux of nitrogen was passed through the solution until 10 mL of a mixture of EtOH and HC(OEt)_3_ distilled out (ca. 45 min). Then the trimethylsilyl chloride (3.0 mL, 23.64 mmol) was added at once and a light blue precipitate formed immediately. The solution was cooled down to room temperature and the precipitate was filtered off, washed with Et_2_O (3 × 10 mL) and then dried to afford 48 mg of **3**-Cl as a slightly green powder (0.157 mmol, 306.79 g/mol). Yield 83%. ^1^H NMR (CDCl_3_, 500 MHz) δ 11.51 (s, 1H, N-C1H1-N,), 8.19 (d, *J =* 8 Hz, 4H, H9, H13, H15, H19), 7.79 (dd, *J =* 3.5 Hz, *J =* 6.3 Hz, 2H, H4, H5), 7.68 (m, 6H, H10, H11, H12, H16, H17, H18), 7.61 (t, *J =* 7.4 Hz, 2H, H3, H6); ^13^C{^1^H}(CDCl_3_, 125 MHz) δ 139.90 (N-C1-N), 130.68 (N-*C*2=*C*7*-N*), 129.39 (C8), 128.80 (*C*4=*C*5), 128.53 (C3), 126.08 (C10), 123.32 (C11), 111.96 (C9); HRMS–ESI (MeOH/H_2_O 1:1): calcd for C_19_H_15_N_2_, 271.12297; found, 271.12281.

**Synthesis of 1,3-di(pyridin-2-yl)-1*H*-benzo[*d*]imidazol-3-ium chloride (4-Cl).** In a 125 mL two neck round-bottomed flask was weighed *N*^1^,*N*^2^-di(pyridin-2-yl)benzene-1,2-diamine (**8**, 300 mg, 1.14 mmol) and triethyl orthoformate (30 mL) was added and a fractional distillation apparatus equipped with a Vigreux column and a thermometer on the head of the latter was connected with the central neck of the flask. The pink suspension was stirred at 145 °C until the suspension became a red solution (10 min), then a small flux of nitrogen was passed through the solution until 20 mL of a mixture of EtOH and HC(OEt)_3_ distilled out (50 min). Then trimethylsilyl chloride (6 mL, 47.27 mmol) was added at once and the solution switched from deep red to blue and a blue precipitate formed immediately. The solution was cooled down to room temperature and the precipitate was filtered off, washed with Et_2_O (3 × 10 mL) and then dried to afford 344 mg of **4**-Cl as a blue powder (1.12 mmol, 308.76 g/mol). Yield 98%. ^1^H NMR (CD_3_CN, 300 MHz) δ 10.07 (s, N-C1H1-N, 1H), 8.84 (dd, *J =* 1.8Hz, *J =* 4.8Hz, 2H, H-12, H-16), 8.48 (dd, *J =* 3.2 Hz, *J =* 6.4 Hz, 2H, H-4, H-5), 8.28 (td, *J =* 1.8 Hz, *J =* 8.1 Hz, 2H, H-10, H-14), 8.03 (dd, *J =* 1.8 Hz, *J =* 8.1Hz, 2H, H-9, H-13), 7.89 (dd, *J* = 3.2 Hz, *J* = 6.4 Hz, 2H, H-3, H-6), 7.77 (td, *J =* 3.9 Hz, *J* = 8.1 Hz, 2H, H-11, H-15); ^13^C{^1^H}(CDCl_3_, 75 MHz) δ 150.0 (s, N-C1-N), 147.52 (C8, C13), 140.73 (C10, C12), 130.96 (N-C2=C7-N), 128.52 (C4=C5), 125.89 (C3, C6), 117.81 (C11, C16), 116.10 (C9, C14); HRMS–ESI (MeOH/H_2_O 1:1): calcd for C_17_H_13_N_4_, 273.11347; found, 273.11308.

**Synthesis of *N*^1^,*N*^2^-diphenylbenzene-1,2-diamine (5).** In a glove-box a 250 mL Schlenk flask was charged with Pd(dba)_2_ (298 mg, 0.52 mmol) and P(*t*-Bu)_3_ (100 mg, 1.04 mmol). Subsequently was added toluene (10 mL) and the solution was stirred for 10 min at room temperature. Then 1,2-dichlorobenzene (0.65 mL, 6.67 mmol) was added followed by aniline addition (1.61 mL, 17.32 mmol) and the *t*-BuONa reagent (1664 mg, 17.32 mmol). An additional amount of toluene (40 mL) was used to rinse the walls of the Schlenk vessel and the solution was then heated at 92 °C. The reaction was monitored by GC–MS and after completion (4 h) to the solution were added AcOEt (100 mL) and water (100 mL). The organic layer was washed with brine and afterwards dried over MgSO_4_ and filtered. After the evaporation of the solvent under reduced pressure we obtained 1547 mg of a deep blue solid (5.94 mmol, *M*_w_ = 260,33). The product has a sky-blue color when in solution, which is due to a small amount of impurities. The ^1^H NMR analysis confirmed that **5** was pure enough to be used for the next synthetic step. Yield 89.1%; GC–MS (EI^+^): 11.074 min [260.3–261.2]; (98%). ^1^H NMR (CDCl_3_, 300 MHz) δ 7.28 (m, 6H), 6.97 (m, 8H), 5.65 (s, 2H, NH); ^13^C{^1^H} NMR (CDCl_3_, 75 MHz) δ 144.8 (s, HN-*C*1=*C*6-NH), 135.0 (s, C7), 129.3 (s, *C*3=*C*4), 122.35 (s, C2), 120.3 (s, C9), 119.4 (s, C10), 116.56 (C8).

**Synthesis of *N*^1^,*N*^2^-bis(2,4,6-trimethylphenyl)benzene-1,2-diamine (6).** Inside a glove box a 250 mL Schlenk flask was charged with Pd(dba)_2_ (23 mg, 0.04 mmol), the P(*t*-Bu)_3_ (9.0 mg, 0.093 mmol) and subsequently toluene (10 mL). The solution was stirred for 10 minutes at room temperature. Then 1,2-dichlorobenzene (0.322 mL, 3.33 mmol) was added followed by 2,4,6-trimethylaniline (1.146 mL, 7.99 mmol) and *t*-BuONa (768 mg, 7.99 mmol) and an additional amount of toluene (40 mL) was used to rinse the walls of the Schlenk tube. The solution was then heated at 115 °C for 15 hours and the solid material that precipitated out of the solution was isolated by suction filtration. The so obtained cake was washed with water and ether and then dried to afford 941 mg of **6** as a grayish powder (2.73 mmol, 344.49 g/mmol). Yield 82%. The ^1^H NMR analysis confirmed that **6** was pure enough to be used for the next step without further purification. MS–EI^+^: 12.485 min [344.5–345.3]; ^1^H NMR (CDCl_3_, 300 MHz) δ 7.025 (s, 4H, H-9, H-11, H-15, H-17), 6.72 (dt, *J* = 3.5 Hz, *J =* 5.7 Hz, 2H, H-4, H-5), 6.37 (dt, *J* = 3.5 Hz, *J =* 5.7 Hz, 2H, H-3, H-6), 5.169 (s broad, 2H, NH), 2.385 (s, 6H, p-CH_3_), 2.26 (s, 12H, o-CH_3_); ^13^C{^1^H}(CDCl_3_, 75 MHz) δ 136.99 (C7, C13), 135.31 (C8, C12, C14, C18), 134.07 (C3, C6), 133.45 (C1, C2), 129.36 (C4, C5), 120.04 (C10, C16), 114.31 (C9, C11, C15, C17), 20.90 (*p*-CH_3_), 18.18 (*o*-CH_3_).

**Synthesis of *N*^1^,*N*^2^-bis[2,6-bis(1-methylethyl)phenyl]benzene-1,2-diamine (7).** Inside a glove box a 250 mL Schlenk flask was charged with Pd(dba)_2_ (98 mg, 0.17 mmol), P(*t*-Bu)_3_ (33 mg, 0.374 mmol), then toluene (20 mL) was added, and the solution was stirred for 5 minutes at room temperature. Addition of 2,6-diisopropylaniline (7.84 mL, 37.4 mmol), *t*-BuONa (4127 mg, 40.8 mmol) and 1,2-dibromobenzene (4.184 mL, 34 mmol), was followed by additional toluene (80 mL). The solution was then heated at 115 °C for 15 hours until a white solid separated from the solution and depositing on the walls of the Schlenk tube. The volume of toluene was reduced to one half and the suspension was filtered through a glass-frit and washed with water (50 mL) and with a mixture of water–methanol (50 mL, 1:2), then the solid was dried under vacuum to afford 8.45 g of **7** as a grey product (19.72 mmol, 428.65 g/mol). The ^1^H NMR analysis established that the product obtained was pure enough to be used for the next step. Yield 58%. MS–EI^+^: 9.97 min [430–431]; ^1^H NMR (CD_2_Cl_2_, 300 MHz) δ 7.31 (m, 6H, H-9, H-10, H-11), 6.66 (dd, *J =* 5.8 Hz, *J =* 3.5 Hz, 2H, H-4, H-5), 6.29 (dd, *J =* 5.8 Hz, *J =* 3.5 Hz, 2H, H-3, H-6), 5.30 (s, 2H, NH), 3.24 (sept., *J =* 6.8 Hz, 4H, CH_3_-C*H*-CH_3_ isopropyl), 1.24 (d, *J =* 6.8 Hz, 12H, C*H_3_*-isopropyl), 1.18 (d, *J =* 6.8 Hz, 12H, C*H_3_*-isopropyl); ^13^C{^1^H} (CD_2_Cl_2_, 75 MHz) δ 145.77 (HN-*C*1=*C*2-NH), 137.25 (NH-*C*7), 137.12 (C8, C12), 126.49 (C10), 124.14 (C9, C11), 120.20 (C4,C5), 114.69 (C6), 28.61 (CH_3_-*C*H-CH_3_, isopropyl), 24.848 (CH_3_, isopropyl), 23.29 (CH_3_, isopropyl).

## Supporting Information

File 1 CIF file of compound **5**.

File 2 NMR and HRMS–ESI analyses.
