# Supplementary material for: A facile synthetic route to benzimidazolium salts bearing bulky aromatic N-substituents
Source: Beilstein J Org Chem. 2015 Sep 17;11:1656–66. doi: 10.3762/bjoc.11.182 (PMC4660899; doi:10.3762/bjoc.11.182)

Supporting Information

for

A facile synthetic route to benzimidazolium salts bearing bulky aromatic N-substituents

Gabriele Grieco [1], Olivier Blacque [1] and Heinz Berke [1]*

Address: [1]Department of Chemistry, University of Zurich, Winterthurerstrasse 190, CH-8057 Zurich, Switzerland, Fax: (+41)-1-635-6802.

Email: Heinz Berke* - hberke@chem.uzh.ch

* Corresponding author

NMR and HRMS–ESI analyses

NMR analyses

1-Cl

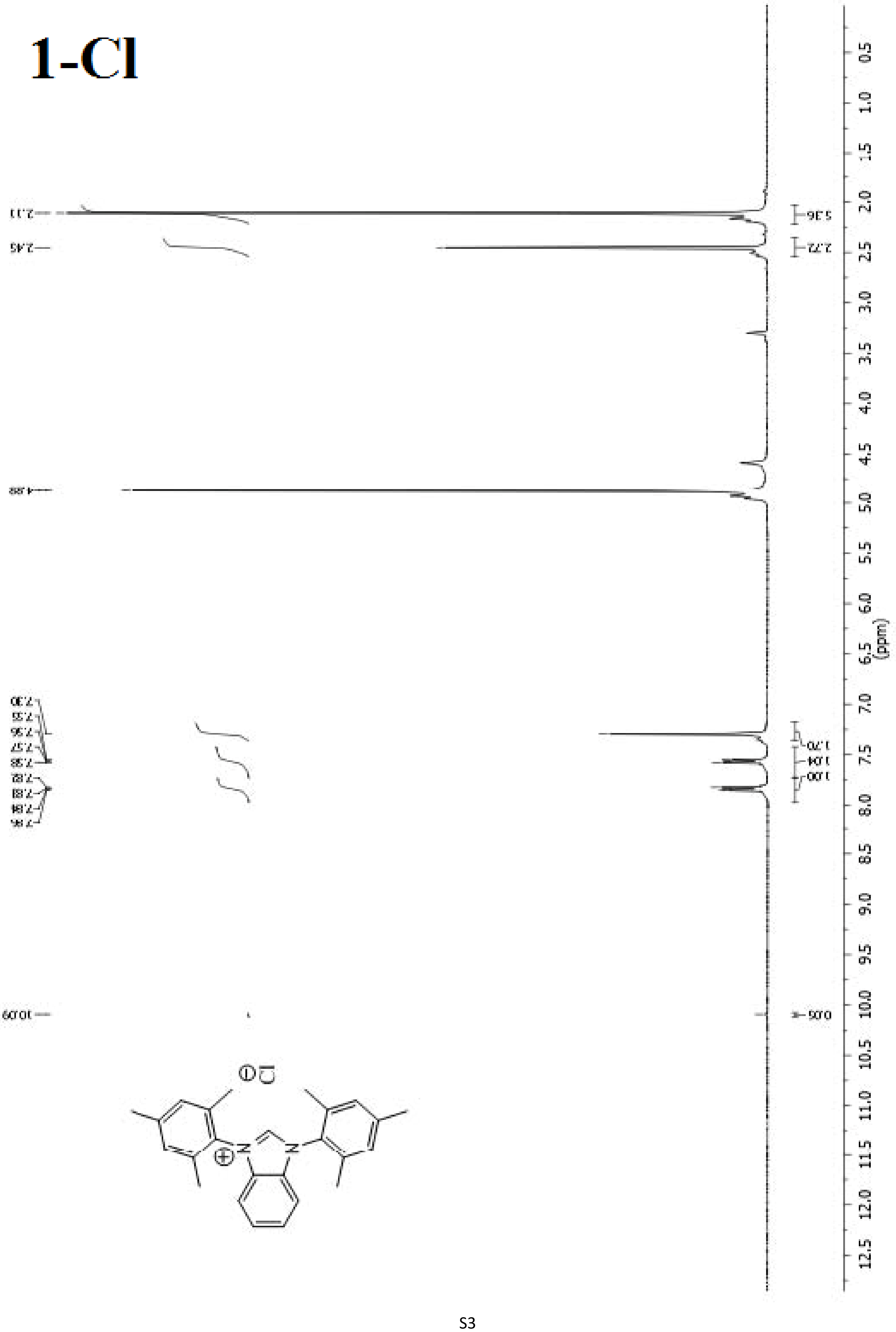

1-Cl

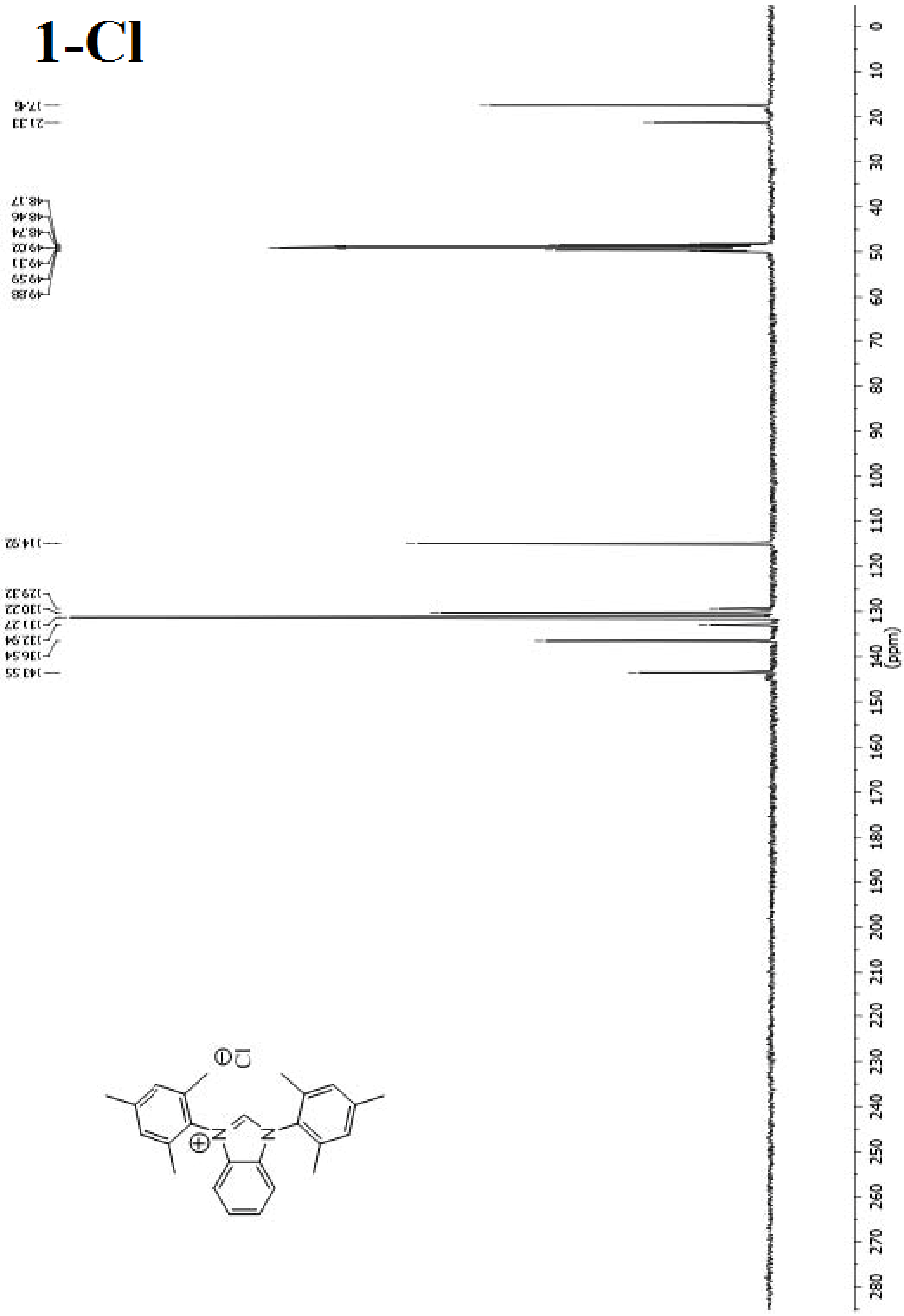
17.46
21.33
48.17
48.46
48.74
49.02
49.31
49.59
49.88
114.92
129.32
130.22
131.27
132.94
136.54
143.55
(ppm)

2-Cl

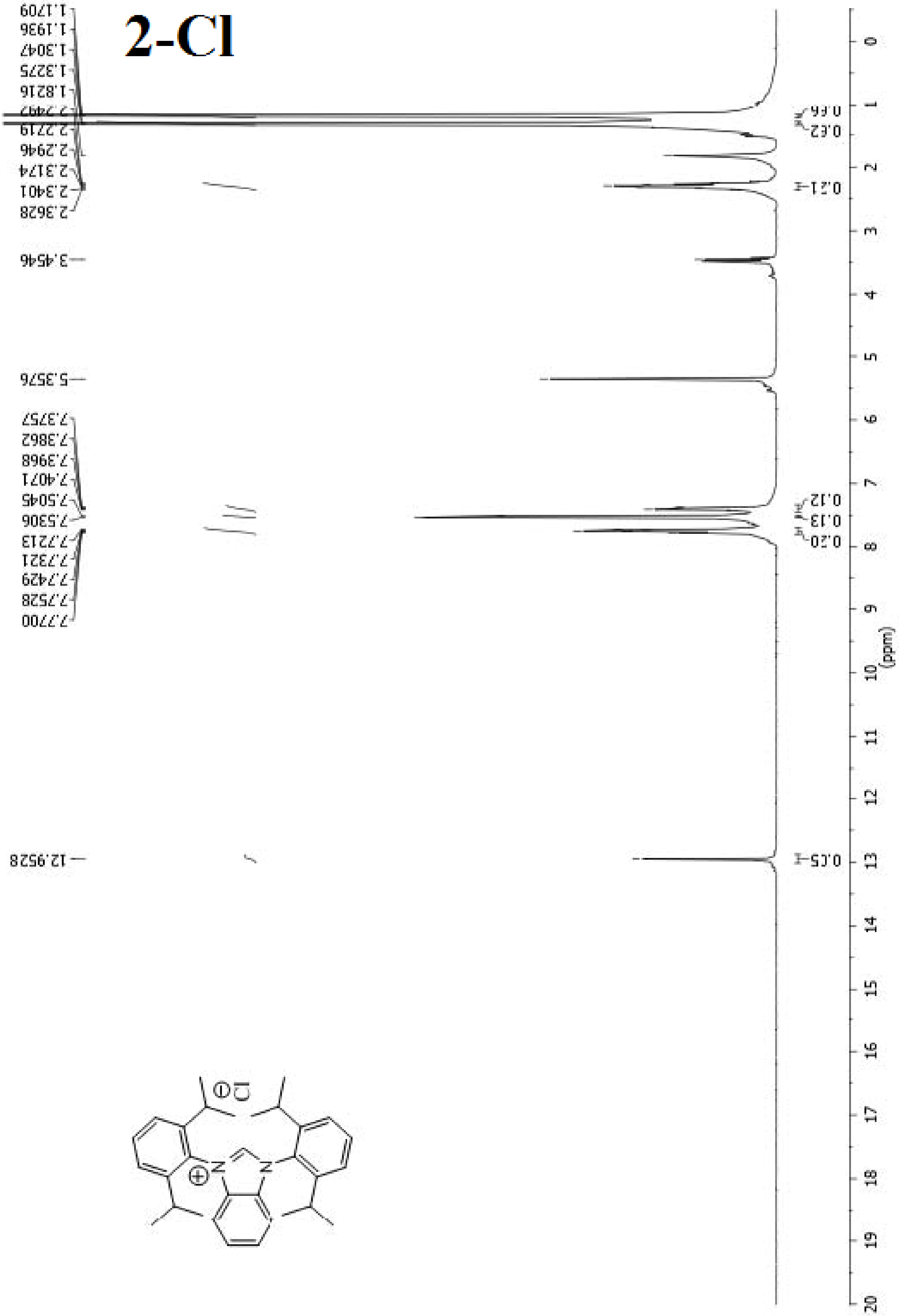

1.1709
1.1936
1.3047
1.3275
1.8216
2.2492
2.2719
2.2946
2.3174
2.3401
2.3628
3.4546
5.3576
7.3757
7.3862
7.3968
7.4071
7.5045
7.5306
7.7213
7.7321
7.7429
7.7528
7.7700
12.9528
(ppm)

2-Cl

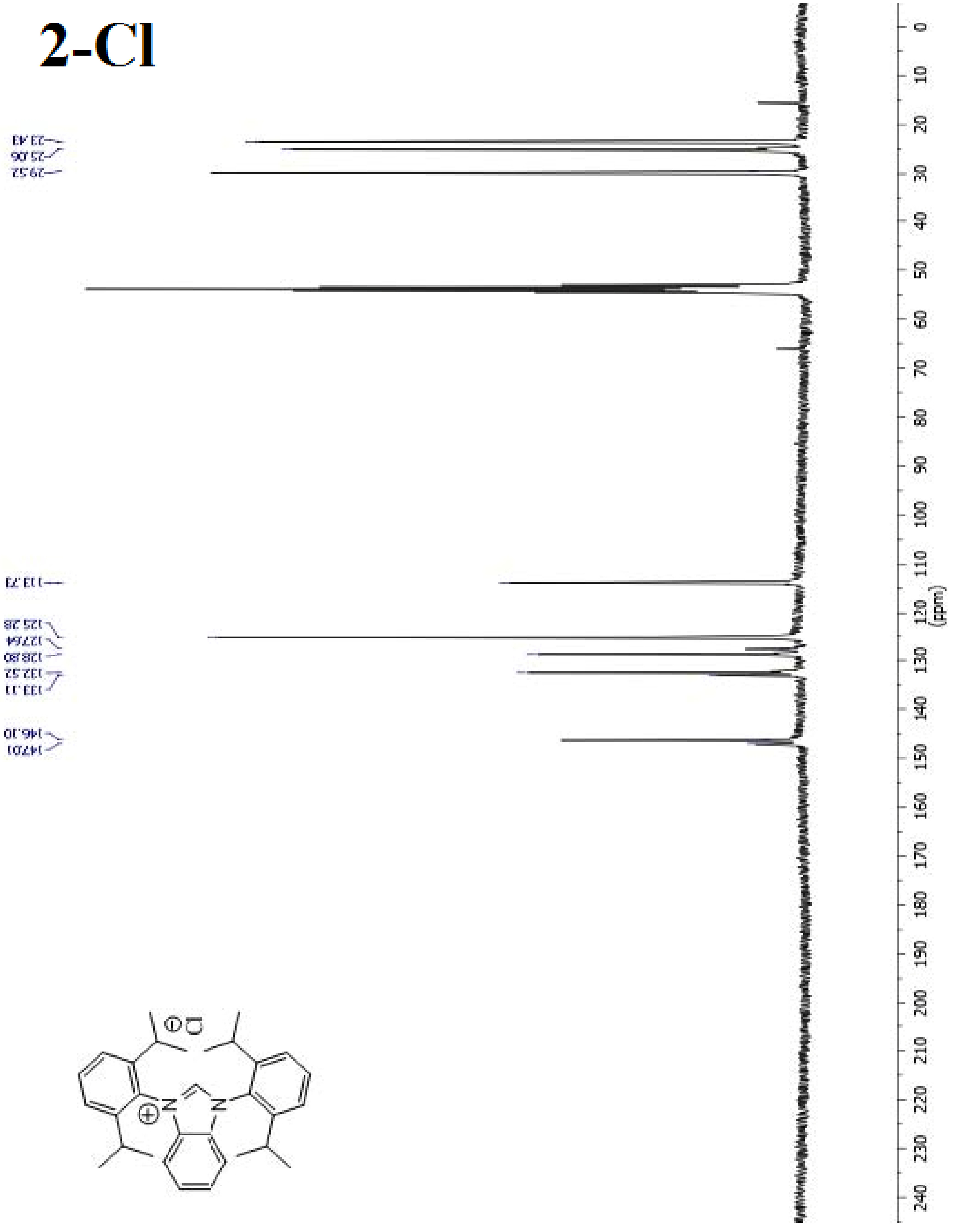
23.43
25.06
29.52
113.73
125.28
127.64
128.80
132.52
133.11
146.10
147.01
(ppm)

3-Cl

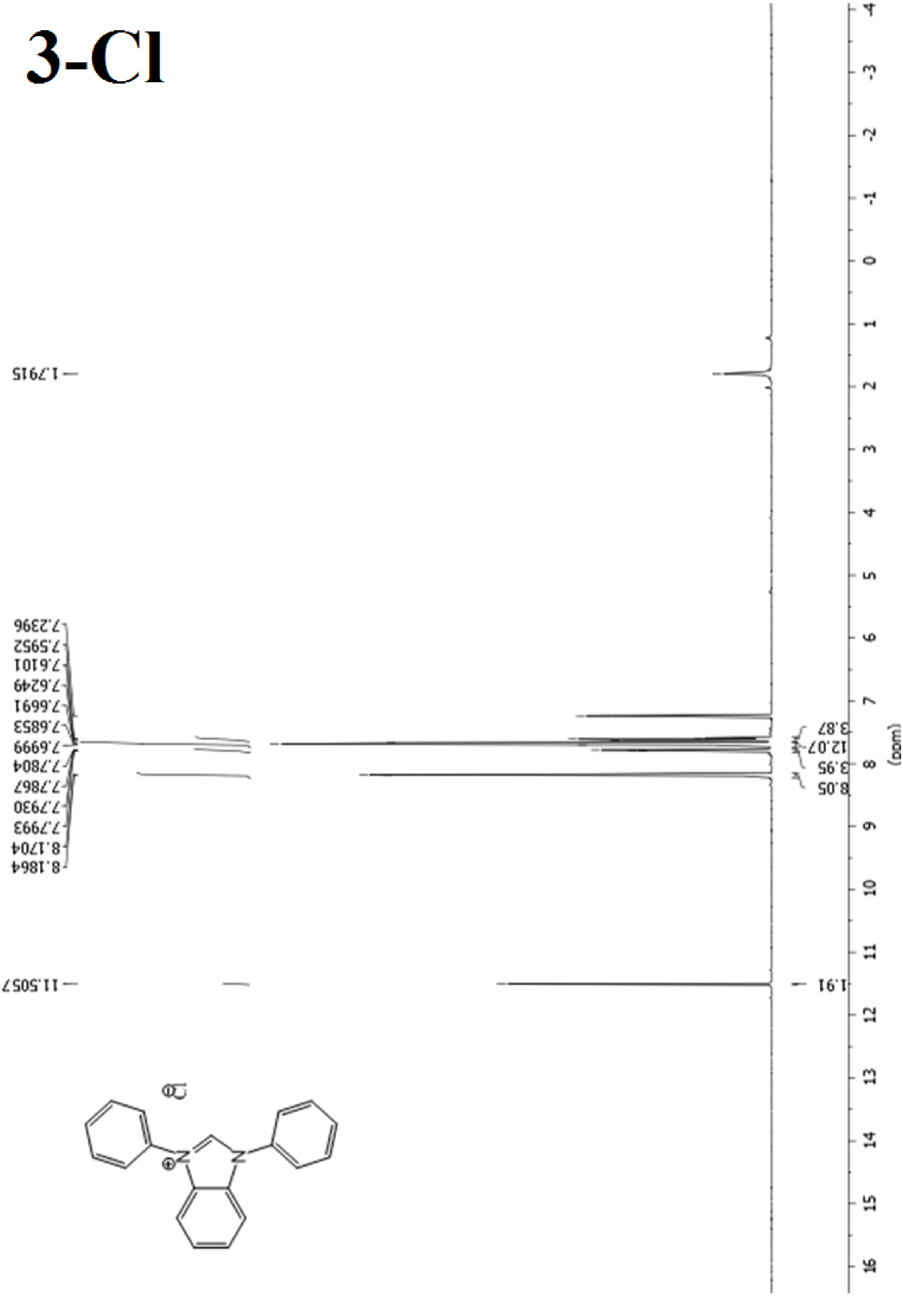

1.7915
7.2396
7.5952
7.6101
7.6249
7.6691
7.6853
7.6999
7.7804
7.7867
7.7930
7.7993
8.1704
8.1864
11.5057
3.87
12.07
3.95
8.05
1.91
(ppm)

3-Cl

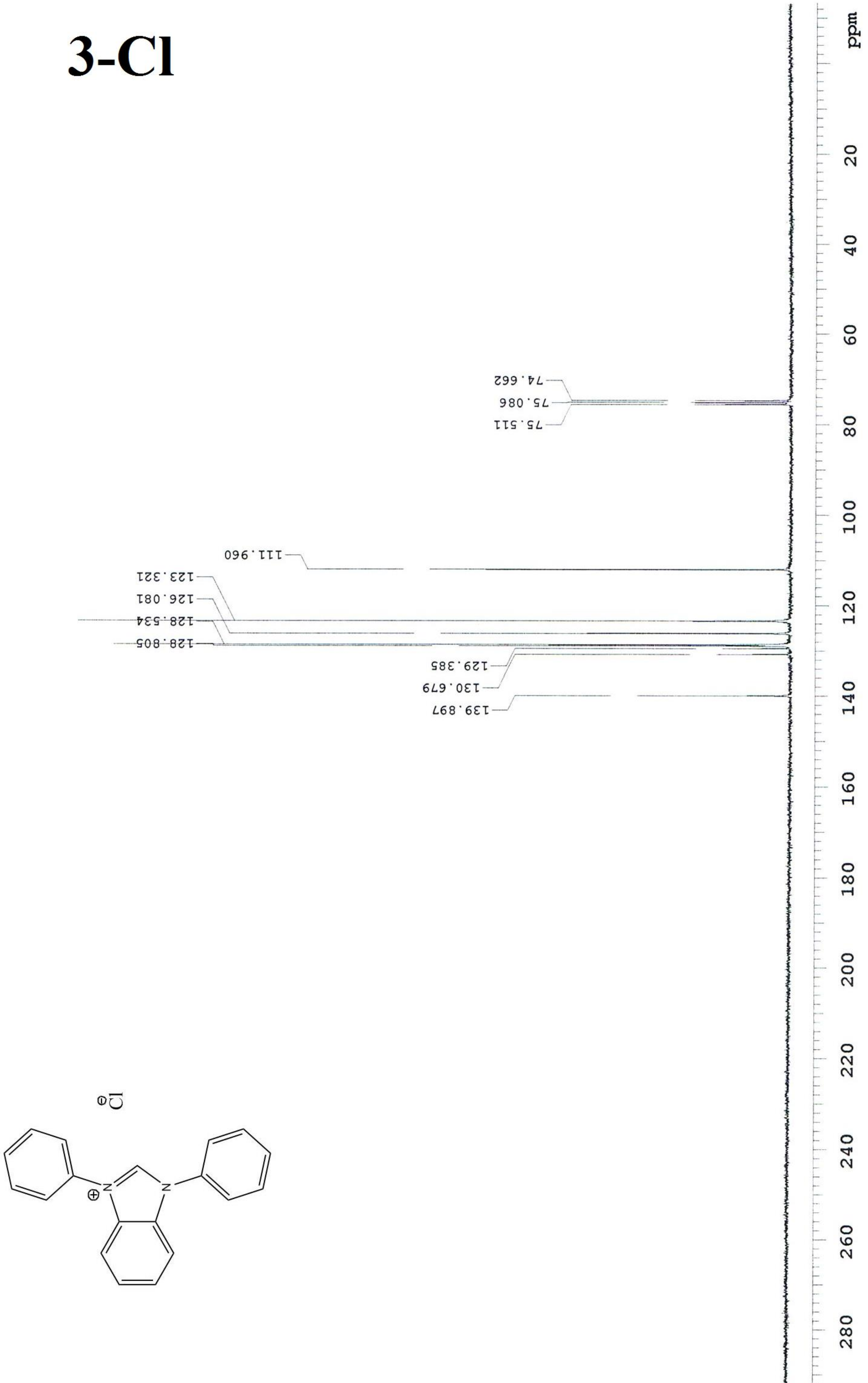
74.662
75.086
75.511
111.960
123.321
126.081
128.534
128.805
129.385
130.679
139.897
ppm
20
40
60
80
100
120
140
160
180
200
220
240
260
280
Cl
N
N

4-Cl

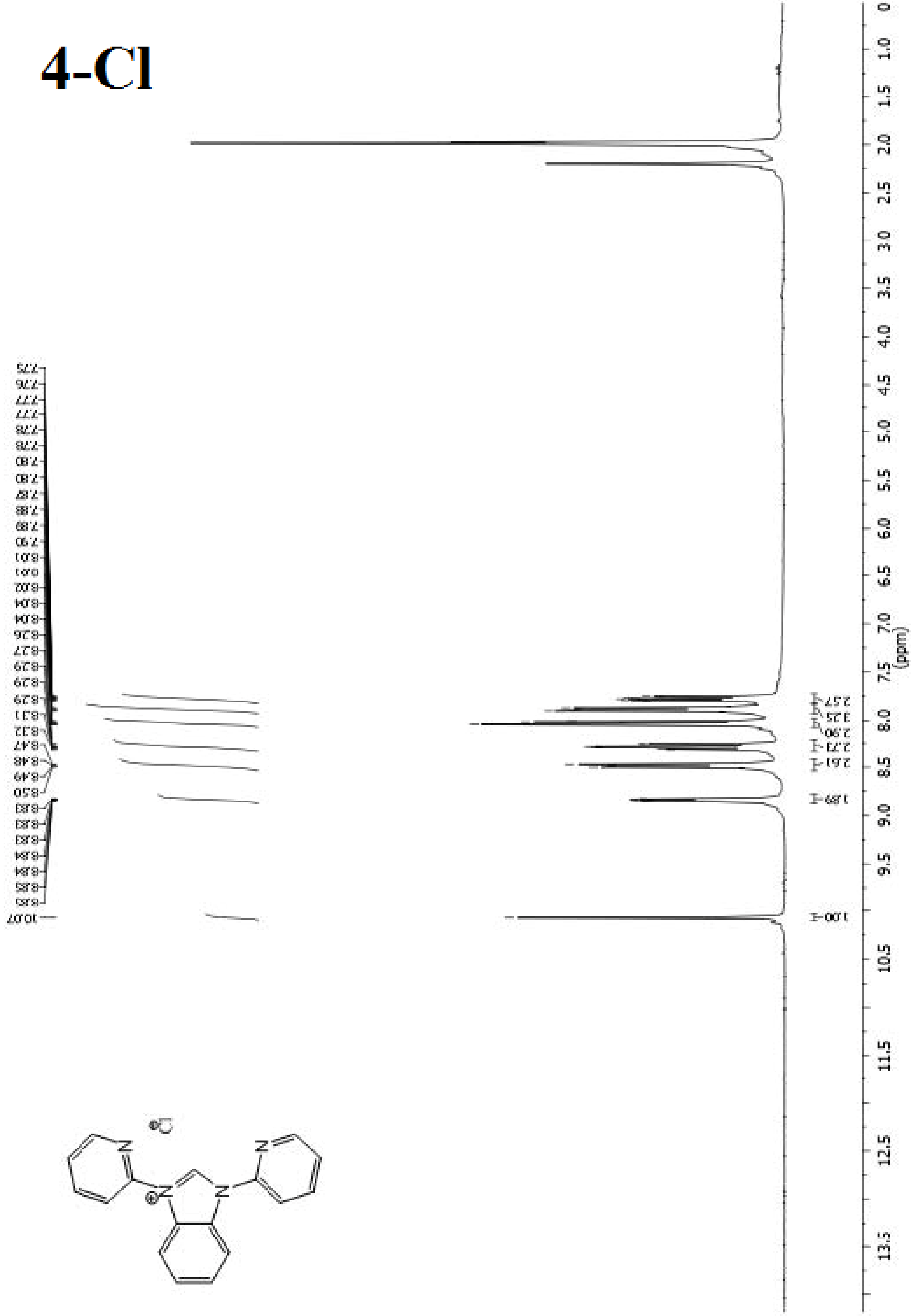

4-Cl

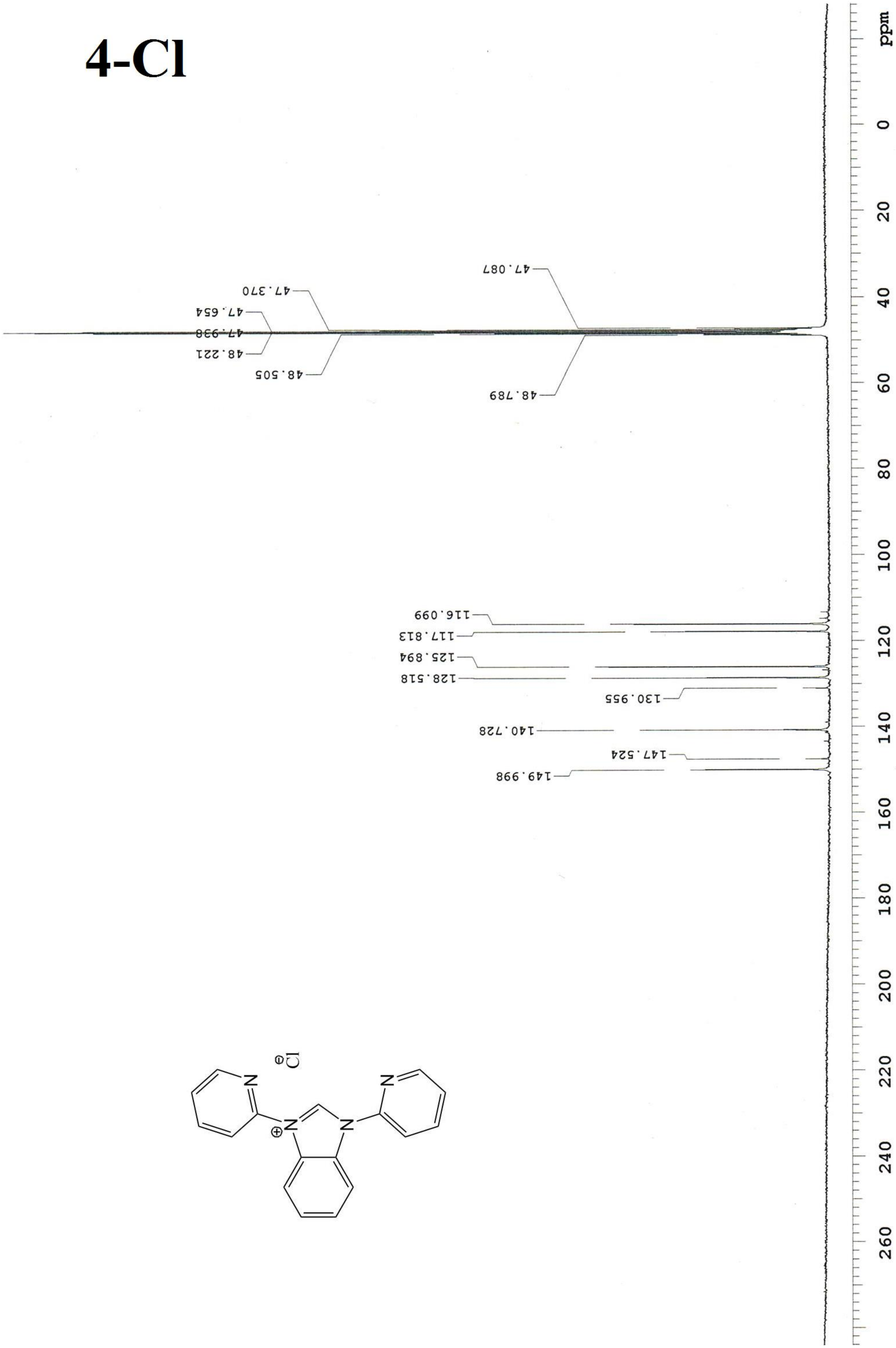

47.087
47.370
47.654
47.938
48.221
48.505
48.789
116.099
117.813
125.894
128.518
130.955
140.728
147.524
149.998
ppm

HRMS–ESI analyses

HR-ESI-MS (Bruker maXis)

Sample Name

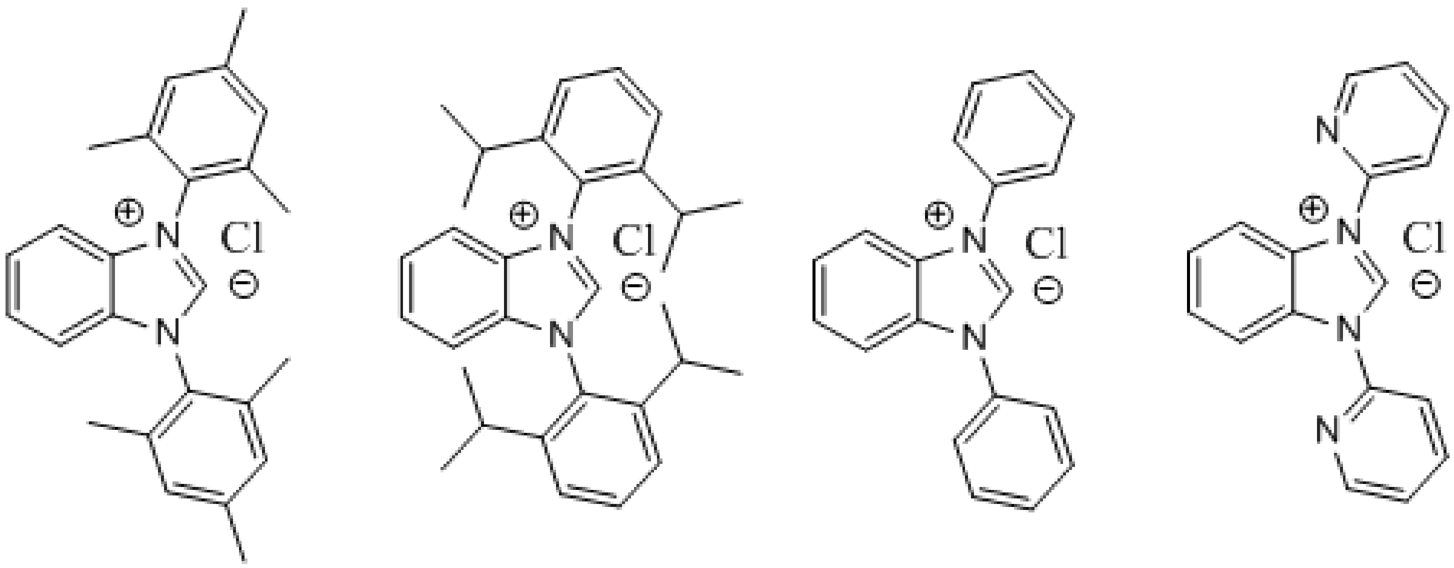

1-Cl = BIMes　　2-Cl = BIPr　　3-Cl = BPh　　4-Cl = BPy

HR-ESI-MS (Bruker maXis)

Analysis Info

Analysis Name	D:\Data\Service\6404alhres.d	Acquisition Date	6/20/2014 9:40:15 AM
Method	tune_low_modified_09_01_14_pos.m	Operator	ust
Sample Name	BIMes	Instrument	maXis 255552.00033
Comment	Solvent: MeOH<br>Client: Grieco		

Acquisition Parameter

Source Type	ESI	Ion Polarity	Positive	Set Nebulizer	0.5 Bar
Scan Begin	50 m/z	Set Capillary	1500 V	Set Dry Heater	180 °C
Scan End	3000 m/z	Set End Plate Offset	-500 V	Set Dry Gas	4.0 l/min

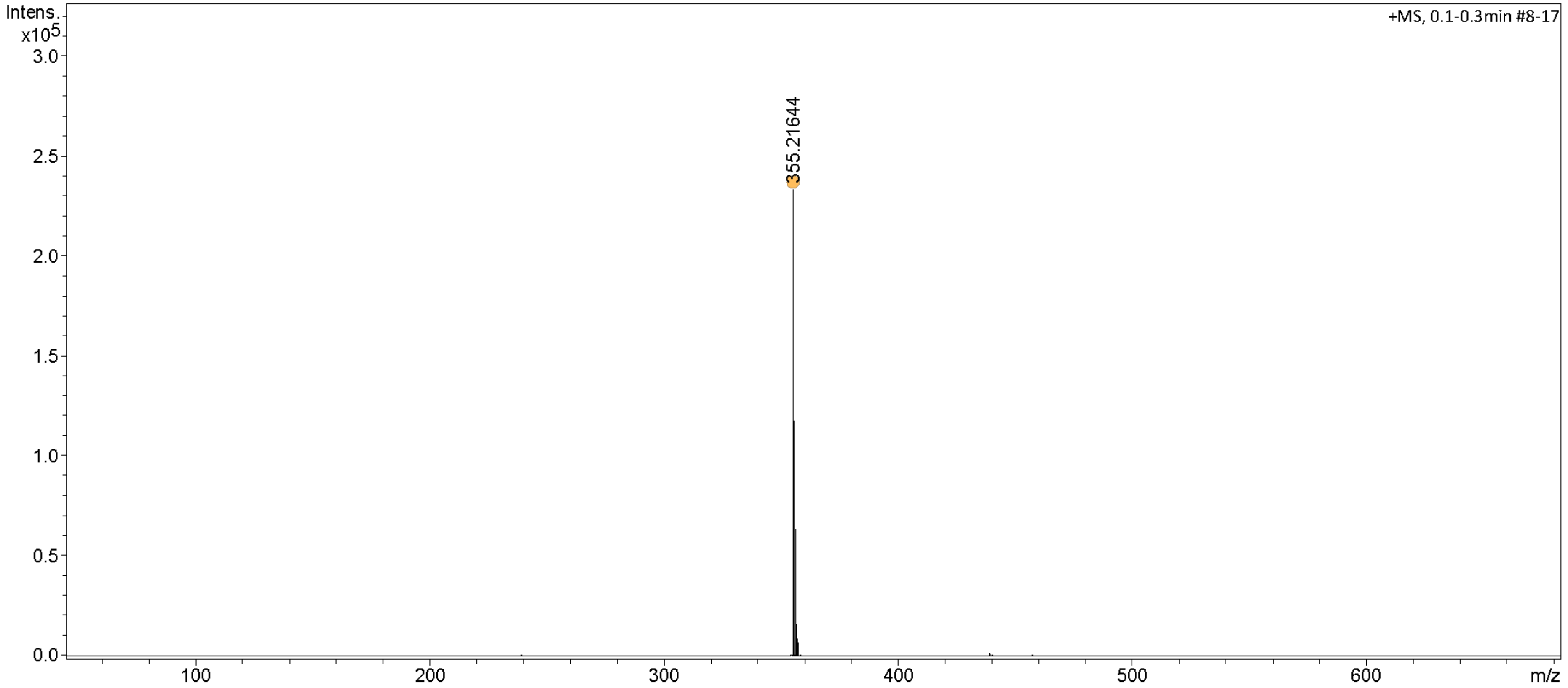

HR-ESI-MS (Bruker maXis)

Meas. m/z	#	Ion Formula	m/z	err [ppm]	mSigma	# mSigma	Score	rdb	e^{-} Conf	N-Rule
355.21644	1	$C_{25}H_{27}N_2$	355.21688	1.23	26.4	1	100.00	13.5	even	ok

HR-ESI-MS (Bruker maXis)

Analysis Info

Analysis Name	D:\Data\Service\6404alhres.d	Acquisition Date	6/20/2014 9:40:15 AM
Method	tune_low_modified_09_01_14_pos.m	Operator	ust
Sample Name	BIMes	Instrument	maXis 255552.00033
Comment	Solvent: MeOH<br>Client: Grieco		

Acquisition Parameter

Source Type	ESI	Ion Polarity	Positive	Set Nebulizer	0.5 Bar
Scan Begin	50 m/z	Set Capillary	1500 V	Set Dry Heater	180 °C
Scan End	3000 m/z	Set End Plate Offset	-500 V	Set Dry Gas	4.0 l/min

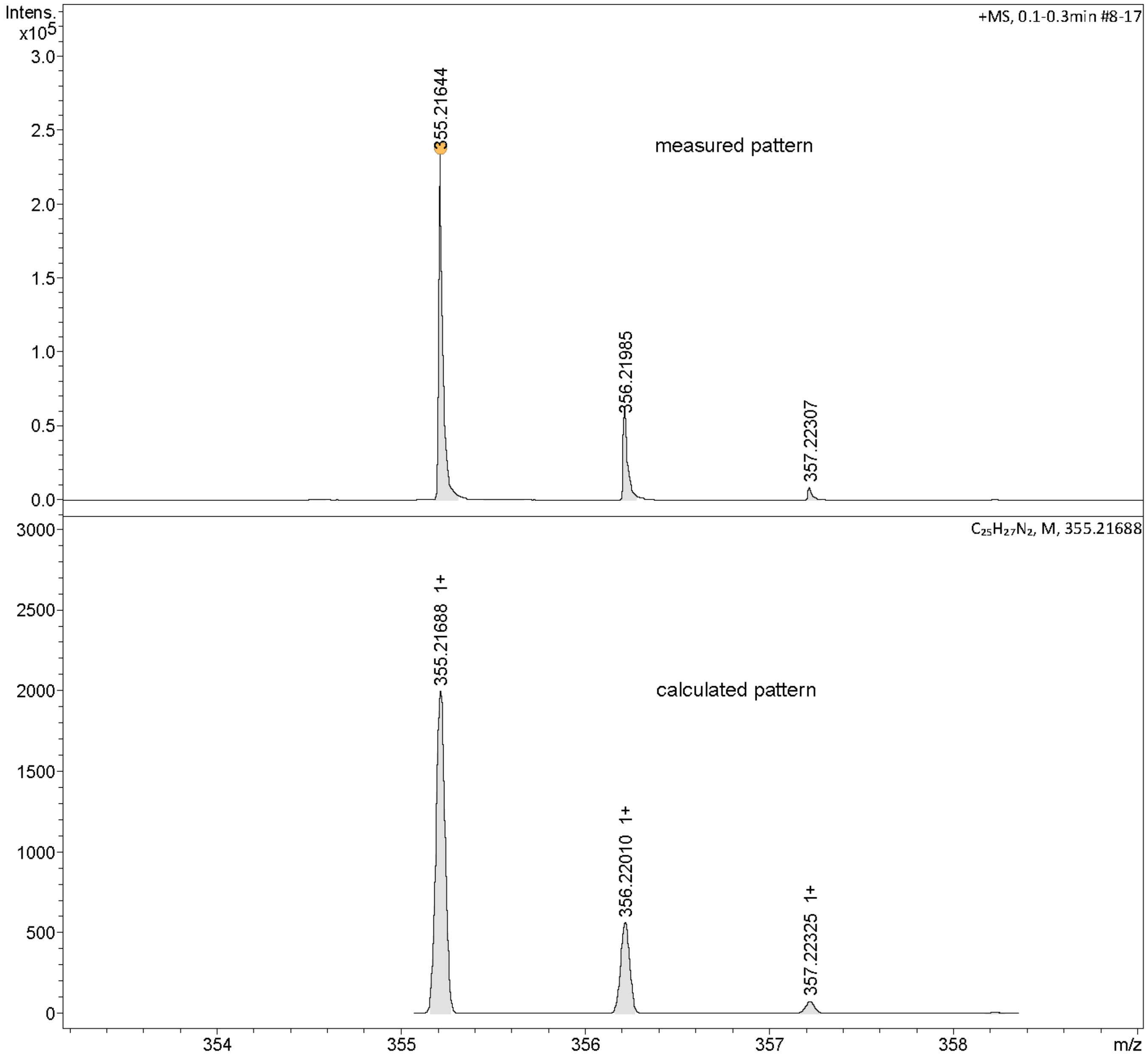

HR-ESI-MS (Bruker maXis)

Analysis Info

Analysis Name	D:\Data\Service\6403alhres.d	Acquisition Date	6/20/2014 9:24:26 AM	
Method	tune_low_modified_09_01_14_pos.m	Operator	ust	
Sample Name	BIPr	Instrument	maXis	255552.00033
Comment	Solvent: MeOH<br>Client: Grieco			

Acquisition Parameter

Source Type	ESI	Ion Polarity	Positive	Set Nebulizer	0.5 Bar
Scan Begin	50 m/z	Set Capillary	1500 V	Set Dry Heater	180 °C
Scan End	3000 m/z	Set End Plate Offset	-500 V	Set Dry Gas	4.0 l/min

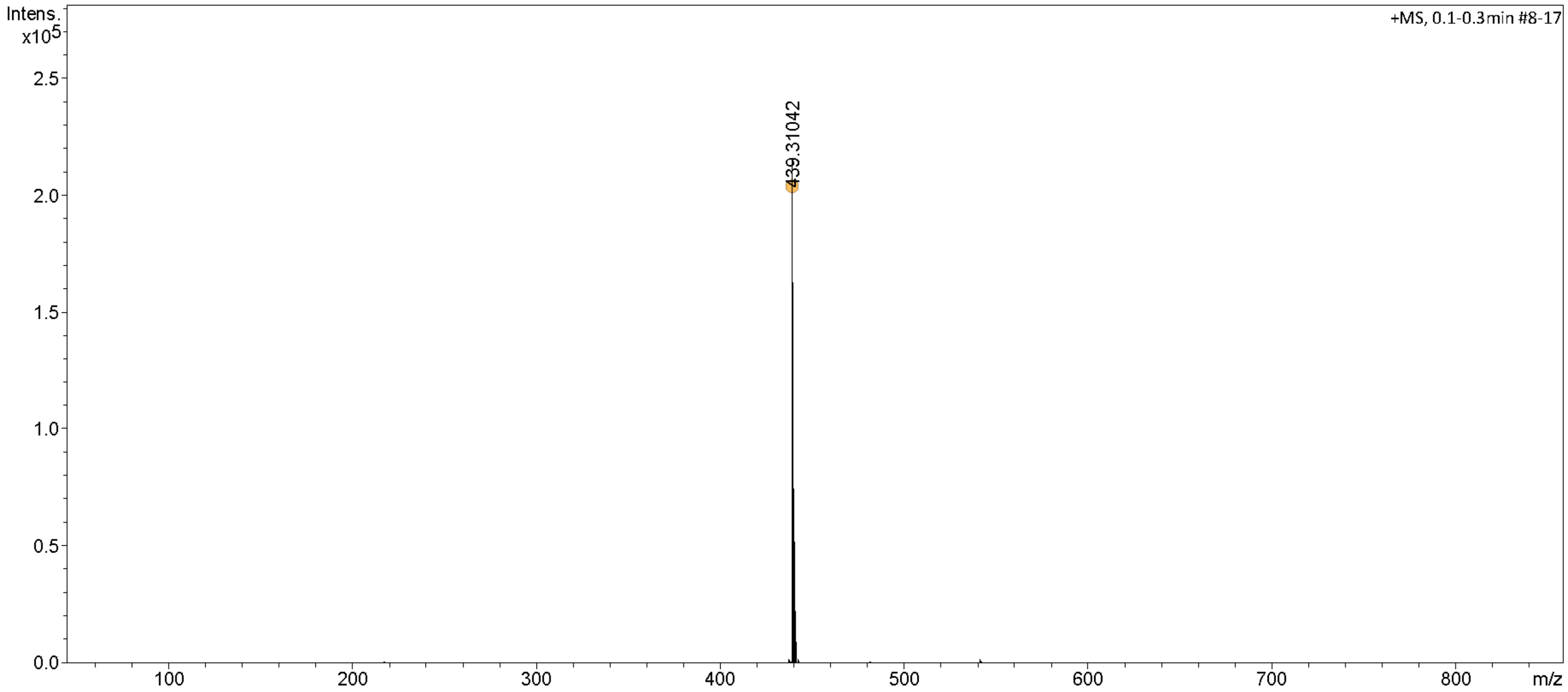

HR-ESI-MS (Bruker maXis)

Meas. m/z	#	Ion Formula	m/z	err [ppm]	mSigma	# mSigma	Score	rdb	e^- Conf	N-Rule
439.31042	1	C31H39N2	439.31078	0.82	13.6	1	100.00	13.5	even	ok
	2	C16H35N14O	439.31128	1.96	83.9	2	9.22	6.5	even	ok
	3	C15H39N10O5	439.30994	-1.08	96.9	3	6.35	1.5	even	ok

HR-ESI-MS (Bruker maXis)

Analysis Info

Analysis Name	D:\Data\Service\6403alhres.d	Acquisition Date	6/20/2014 9:24:26 AM
Method	tune_low_modified_09_01_14_pos.m	Operator	ust
Sample Name	BIPr	Instrument	maXis 255552.00033
Comment	Solvent: MeOH<br>Client: Grieco		

Acquisition Parameter

Source Type	ESI	Ion Polarity	Positive	Set Nebulizer	0.5 Bar
Scan Begin	50 m/z	Set Capillary	1500 V	Set Dry Heater	180 °C
Scan End	3000 m/z	Set End Plate Offset	-500 V	Set Dry Gas	4.0 l/min

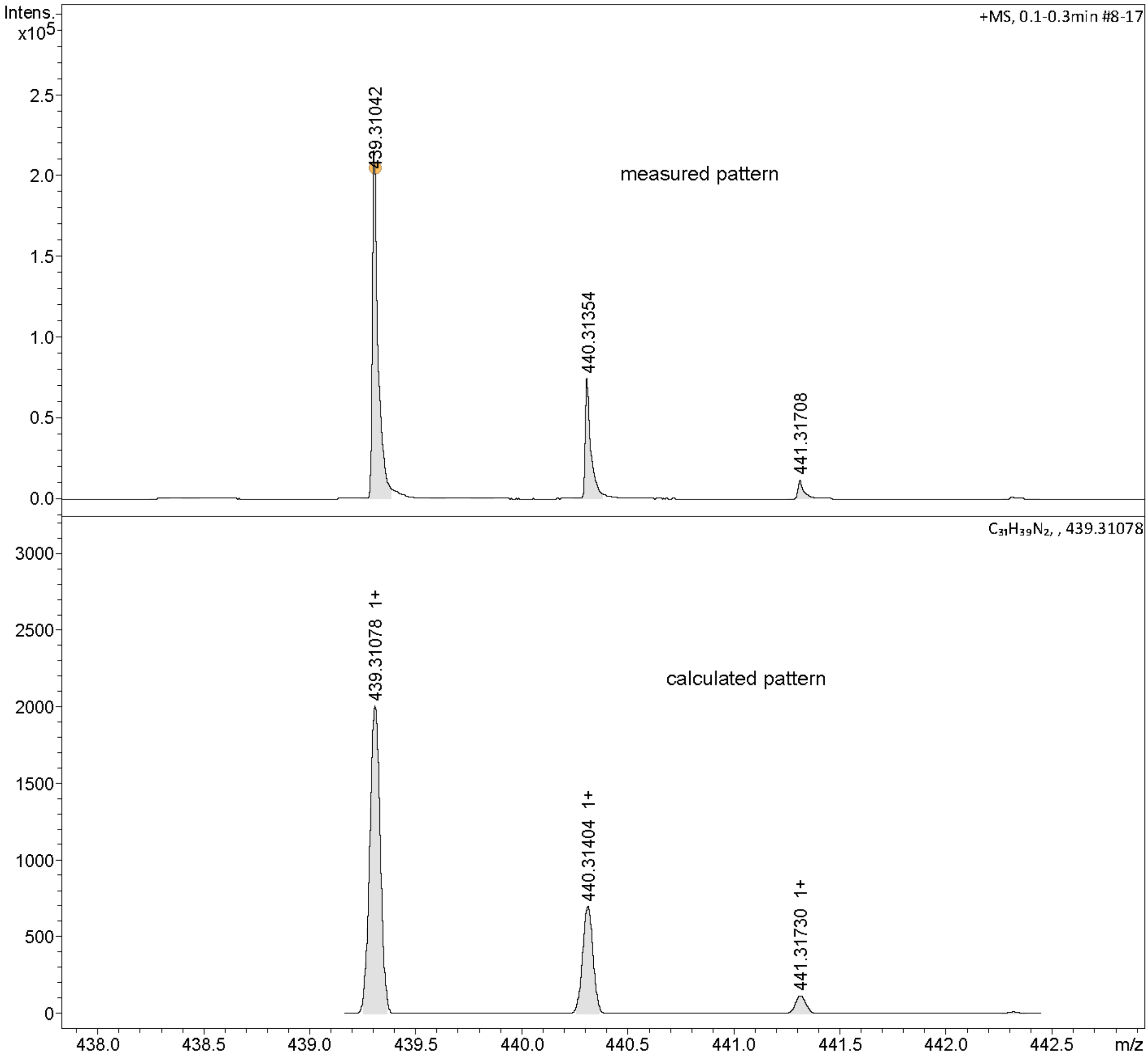

HR-ESI-MS (Bruker maXis)

Analysis Info

Analysis Name	D:\Data\Service\6513alhres.d	Acquisition Date	7/7/2014 5:02:28 PM
Method	tune_low_modified_09_01_14_pos.m	Operator	ust
Sample Name	BPh	Instrument	maXis 255552.00033
Comment	Solvent: MeOH/H2O 1:1<br>Client: Grieco		

Acquisition Parameter

Source Type	ESI	Ion Polarity	Positive	Set Nebulizer	0.5 Bar
Scan Begin	50 m/z	Set Capillary	1000 V	Set Dry Heater	180 °C
Scan End	3000 m/z	Set End Plate Offset	-500 V	Set Dry Gas	4.0 l/min

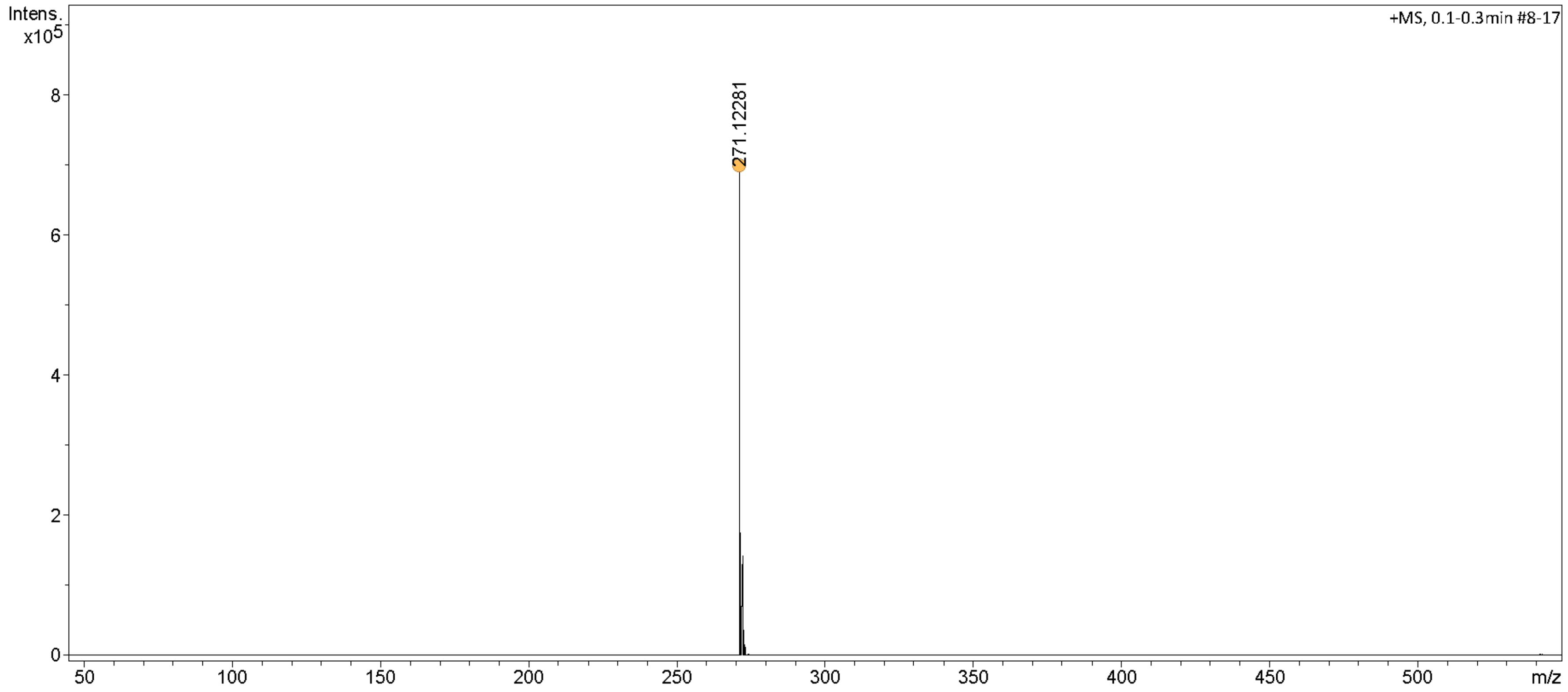

HR-ESI-MS (Bruker maXis)

Analysis Info

Analysis Name	D:\Data\Service\6513alhres.d	Acquisition Date	7/7/2014 5:02:28 PM
Method	tune_low_modified_09_01_14_pos.m	Operator	ust
Sample Name	BPh	Instrument	maXis 255552.00033
Comment	Solvent: MeOH/H2O 1:1<br>Client: Grieco		

Acquisition Parameter

Source Type	ESI	Ion Polarity	Positive	Set Nebulizer	0.5 Bar
Scan Begin	50 m/z	Set Capillary	1000 V	Set Dry Heater	180 °C
Scan End	3000 m/z	Set End Plate Offset	-500 V	Set Dry Gas	4.0 l/min

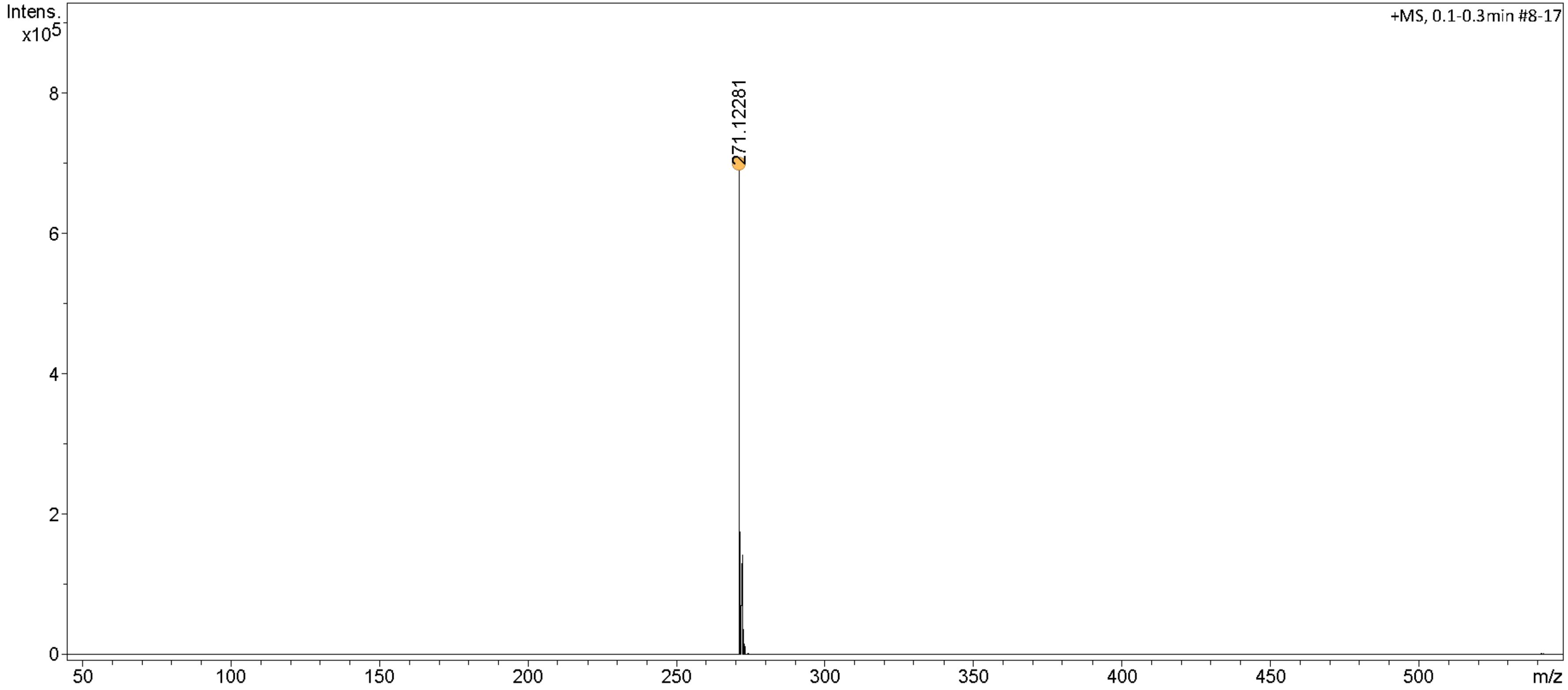

HR-ESI-MS (Bruker maXis)

Analysis Info

Analysis Name	D:\Data\Service\6513alhres.d	Acquisition Date	7/7/2014 5:02:28 PM
Method	tune_low_modified_09_01_14_pos.m	Operator	ust
Sample Name	BPh	Instrument	maXis 255552.00033
Comment	Solvent: MeOH/H2O 1:1<br>Client: Grieco		

Acquisition Parameter

Source Type	ESI	Ion Polarity	Positive	Set Nebulizer	0.5 Bar
Scan Begin	50 m/z	Set Capillary	1000 V	Set Dry Heater	180 °C
Scan End	3000 m/z	Set End Plate Offset	-500 V	Set Dry Gas	4.0 l/min

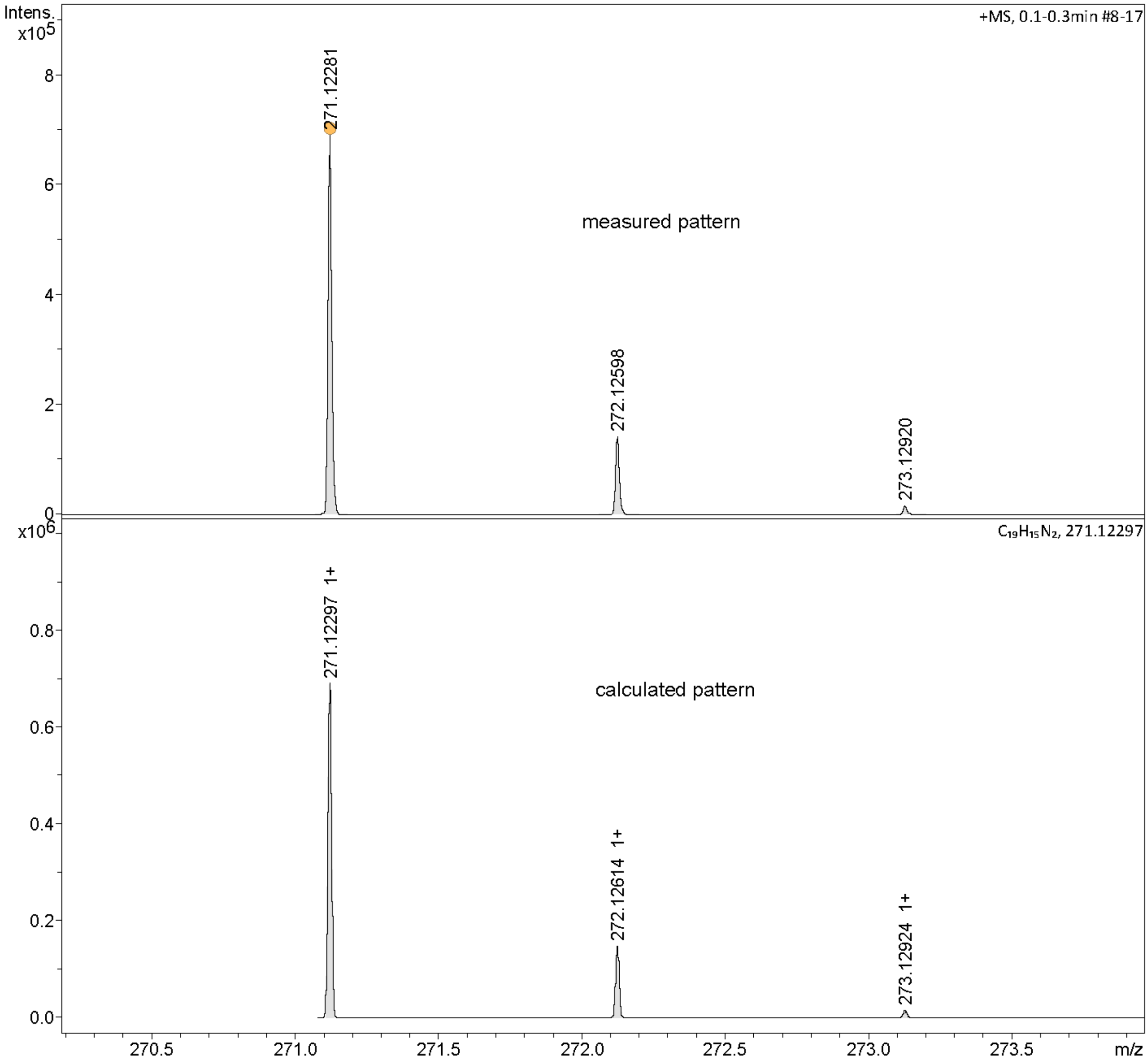

HR-ESI-MS (Bruker maXis)

Analysis Info

Analysis Name	D:\Data\Service\6514alhres.d	Acquisition Date	7/7/2014 5:22:43 PM
Method	tune_low_modified_09_01_14_pos.m	Operator	ust
Sample Name	BPy	Instrument	maXis 255552.00033
Comment	Solvent: MeOH/H2O 1:1<br>Client: Grieco		

Acquisition Parameter

Source Type	ESI	Ion Polarity	Positive	Set Nebulizer	0.5 Bar
Scan Begin	50 m/z	Set Capillary	1500 V	Set Dry Heater	180 °C
Scan End	3000 m/z	Set End Plate Offset	-500 V	Set Dry Gas	4.0 l/min

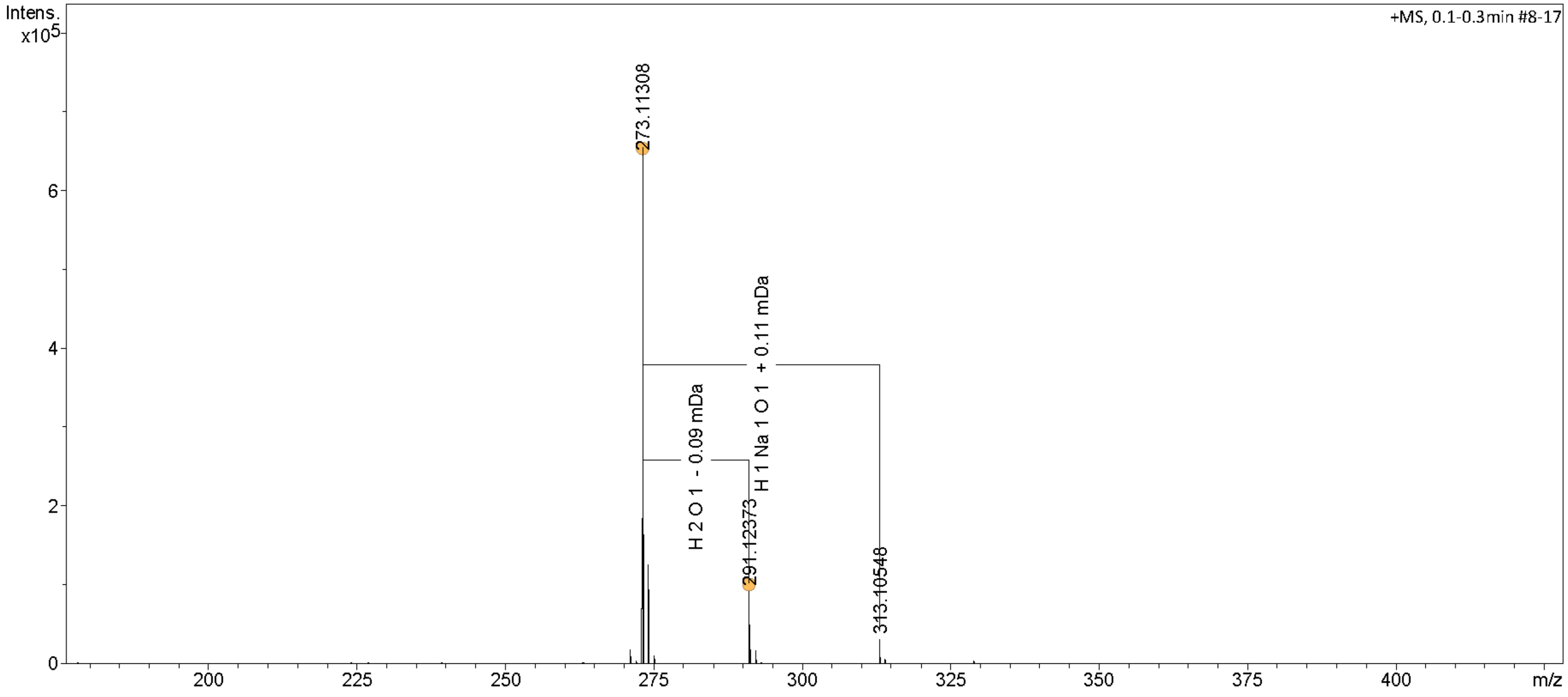

HR-ESI-MS (Bruker maXis)

Analysis Info

Analysis Name	D:\Data\Service\6514alhres.d	Acquisition Date	7/7/2014 5:22:43 PM
Method	tune_low_modified_09_01_14_pos.m	Operator	ust
Sample Name	BPy	Instrument	maXis 255552.00033
Comment	Solvent: MeOH/H2O 1:1<br>Client: Grieco		

Acquisition Parameter

Source Type	ESI	Ion Polarity	Positive	Set Nebulizer	0.5 Bar
Scan Begin	50 m/z	Set Capillary	1500 V	Set Dry Heater	180 °C
Scan End	3000 m/z	Set End Plate Offset	-500 V	Set Dry Gas	4.0 l/min

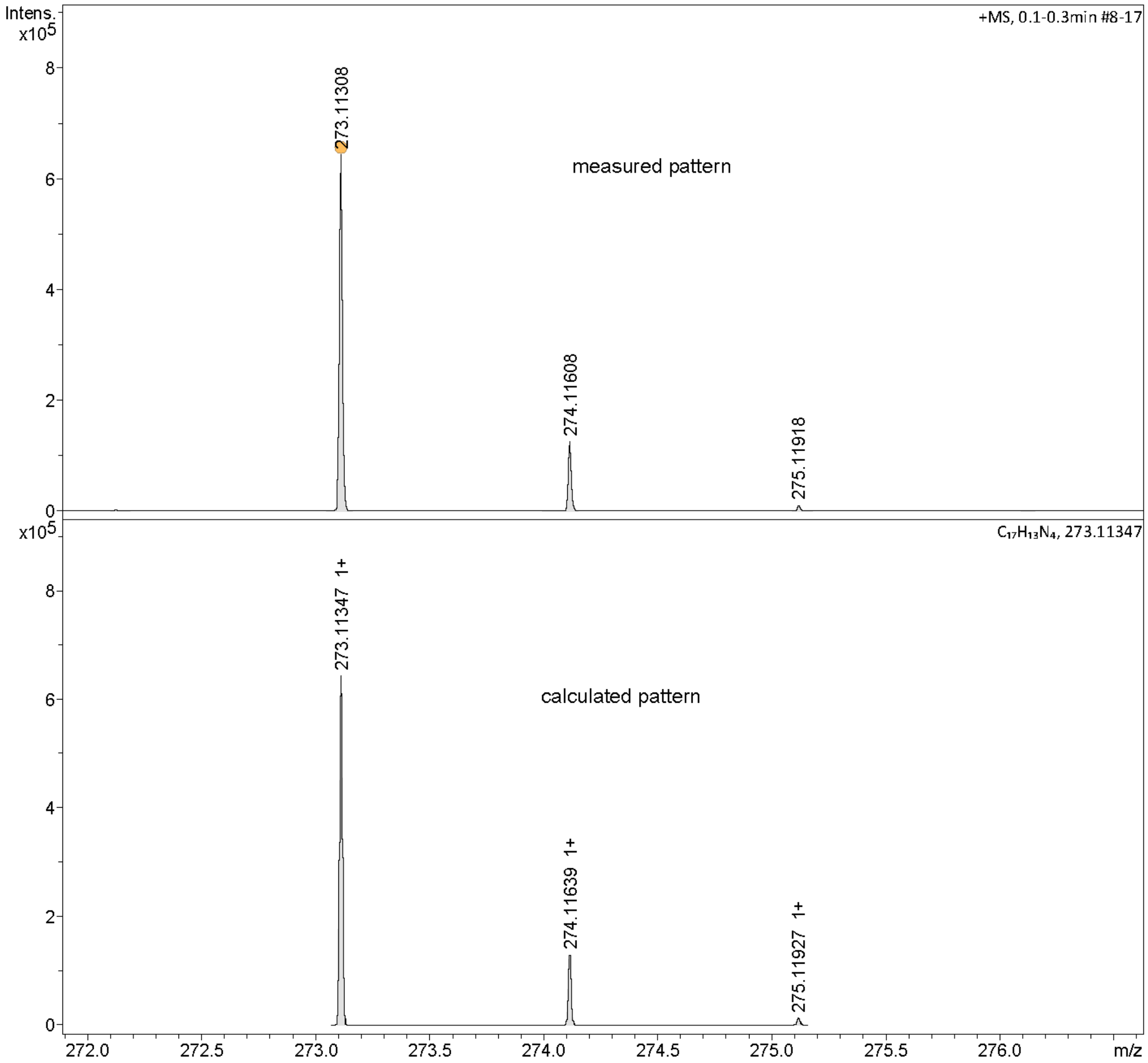

Supplement: File 2 — NMR and HRMS–ESI analyses. [file Beilstein_J_Org_Chem-11-1656-s002.pdf]
